# Quality of Life in Caregivers of Patients with Schizophrenia: A Systematic Review of the Impact of Sociodemographic, Clinical, and Psychological Factors

**DOI:** 10.3390/bs15050684

**Published:** 2025-05-17

**Authors:** Corina Gagiu, Vlad Dionisie, Mihnea Costin Manea, Anca Covaliu, Ana Diana Vlad, Ancuta Elena Tupu, Mirela Manea

**Affiliations:** 1 Department of Psychiatry and Psychology, “Carol Davila” University of Medicine and Pharmacy, 020021 Bucharest, Romania; 2 Department of Adult Psychiatry, “Prof. Dr. Alexandru Obregia” Clinical Hospital of Psychiatry, 041914 Bucharest, Romania; 3 Department of Scientific Research Methodology, Faculty of Medicine and Pharmacy, “Dunarea de Jos” University of Galati, 800008 Galati, Romania

**Keywords:** schizophrenia, caregiver, family, quality of life, life satisfaction, well-being, burden

## Abstract

Caregiving for a patient with schizophrenia (PwS) imposes a high burden on caregivers and often affects their quality of life. This systematic review aims to synthesize the current evidence on the sociodemographic and psychological factors of caregivers, as well as patient-related sociodemographic and clinical factors, that may influence caregivers’ QoL. The review was conducted following the Preferred Reporting Items for Systematic Reviews and Meta-Analyses (PRISMA) guidelines. A comprehensive literature search was performed in three major databases—PubMed/Medline, SCOPUS, and Web of Science—to identify original studies examining informal caregivers of PwS and assessing the relationship between caregivers’ QoL and various sociodemographic, psychological, or clinical factors. Methodological quality appraisal was performed using the Joanna Briggs Institute checklist. In total, 31 studies were included in the review and discussed at length. Lower QoL was associated with unemployment, older age, female gender, financial difficulties, being unmarried, and lower education. Additionally, increased schizophrenia symptom severity, higher caregiver burden, and elevated levels of depression and anxiety may negatively influence caregivers’ QoL. Given these findings, future research should focus on developing tailored interventions to improve caregivers’ QoL. Addressing these modifiable risk factors through targeted support programs and policies could significantly enhance caregivers’ QoL.

## 1. Introduction

Schizophrenia is a chronic, severe, and debilitating mental disorder characterized by a range of signs and symptoms, including hallucinations, delusions, disorganized thinking and behavior, and negative symptoms such as social withdrawal and anhedonia (Kumar et al., 2017; Lungu et al., 2023; Tandon et al., 2008). Schizophrenia typically has its onset in early adulthood and has a global prevalence of approximately 1% (1 in 300 people worldwide is affected by schizophrenia) (Gogtay et al., 2011; Lungu et al., 2023; Saha et al., 2005). This early onset significantly impacts patients, influencing multiple aspects of their lives, including personal, social, educational, and professional development. It also leads to financial and familial difficulties (Addington & Heinssen, 2012; Arango et al., 2022). Moreover, numerous studies have shown that schizophrenia is associated with high rates of comorbid conditions such as depression, anxiety, and substance abuse (Tsai & Rosenheck, 2013), which complicate treatment and increase the overall burden of care (Buckley et al., 2009).

In recent years, the treatment of PwS has shifted from institutionalization to community-based care, which has led families to take on responsibilities that were once handled by hospitals (Pan et al., 2024). A caregiver is any individual who provides care to a family member, partner, or friend suffering from a debilitating chronic mental health condition such as schizophrenia. Caregivers are typically unpaid and assume responsibility altruistically, supporting patients in their daily lives. Caregivers play a vital role in the healthcare system, compensating for the lack of resources and reducing pressure on medical services as they manage relapse episodes and help prevent violence and self-harm, for example (Liu, 2024). Caregiving for PwS can lead to various personal experiences. For instance, they often experience stress, anxiety, and depression due to ongoing responsibilities and uncertainty regarding the disorder’s progression. Many report feelings of social isolation and difficulties maintaining social and professional relationships. The financial costs associated with care, including medication and medical visits, can be overwhelming, especially for low-income families (Cleary et al., 2020; Shiraishi & Reilly, 2019). The relationship with the patient can also become strained, especially during episodes of relapse (Kulhara, 2012). Despite these challenges, some caregivers find meaning and satisfaction in their roles, describing themselves as more empathetic and patient, with different perspectives on life priorities and a sense of inner strength, fulfillment, and gratitude when they succeed in helping the patient (Cleary et al., 2020; Kulhara, 2012; Shiraishi & Reilly, 2019).

QoL is generally defined as an indicator of an individual’s physical, mental, emotional, and social well-being. The WHO describes it as “the individual’s perception of their position in life in the context of the culture and value system in which they live, in relation to their goals, expectations, standards, and concerns” (The WHOQOL Group, 1998). Numerous studies have shown that caregivers of PwS have a marked decline in their QoL compared to the general population. The areas most commonly found to decline were the mental/psychological, physical, social, and environmental components, which suggests that caregiving for a PwS has a wide-ranging impact on QoL (Cohen et al., 2022; Hayes et al., 2015; Margetić et al., 2013; Kojima et al., 2024; Csoboth et al., 2015). The QoL of caregivers is thought to be influenced by a complex interplay of miscellaneous factors, including aspects related to both the patient and the caregiver. It was found that overall QoL was negatively affected by higher symptom intensity in patients (measured with PANSS) with physical and psychological burdens and daily life domains being particularly impacted (Caqueo-Urízar et al., 2016b). Other studies have demonstrated that the chronicity of the illness leads to a gradual decline in the QoL of caregivers, chronic fatigue, and deterioration in their mental health (Awad & Voruganti, 2008; Chien et al., 2007; Grandón et al., 2008; Lee et al., 2006; Ochoa et al., 2008). Moreover, social support can alleviate caregiver stress and improve their QoL (Foldemo et al., 2005). Additionally, financial problems generate concern among caregivers due to expenses related to treatment, therapy, and hospitalizations (van Os & Kapur, 2009). In this regard, there is a difference between developed and developing countries (Bradley et al., 2006; Gutiérrez-Maldonado et al., 2005; Ohaeri, 2001). For instance, caregivers from Nigeria often reported financial strain due to the lack of government support and the need to cover treatment and hospitalization out-of-pocket (Ohaeri, 2001). Chilean caregivers had lower QoL in the physical domain in comparison to their French counterparts, which was largely explained by the limited access to health and economic resources (Boyer et al., 2012). Family dynamics have also been affected due to numerous disagreements, conflicts, and even violence within the family unit (Thara et al., 2003). While tailored support according to caregivers’ needs and psycho-social support and psychoeducation are recommended to sustain good life satisfaction, the existing comprehensive guidelines remain limited to a few countries (e.g., United Kingdom, Canada) and incomplete. Moreover, studies exploring the needs of caregivers of people with mental illness revealed that support for caregivers in the areas of information and education about schizophrenia, mental hygiene, self-help groups, and healthcare services is limited (Issac et al., 2022; National Collaborating Centre for Mental Health (UK), 2014).

By systematically analyzing the existing literature on this topic, this review seeks to identify the sociodemographic, clinical, and psychological aspects of both patients and caregivers that are shown to impact QoL in caregivers. In addition, this review aims to provide insights that can inform healthcare strategies and interventions aimed at alleviating the strain on caregivers and improving their well-being. As far as we are aware, this is the first systematic review that specifically investigates these factors that may affect caregivers’ QoL.

## 2. Materials and Methods

In the preparation of the present systematic review, we adhered to the PRISMA guidelines (Page et al., 2021). The PRIMSA 2020 checklist is provided in the Supplementary Materials (Table S1). The protocol of this review was registered in PROSPERO with the registration number CRD42024617926.

To establish the research question and develop the objectives and eligibility criteria, an adapted PICO framework was used (Melnyk & Fineout-Overholt, 2023). Therefore, this systematic review is based on the following question: “In caregivers of patients with schizophrenia (P), what are the effects of socio-demographic, clinical and psychological factors (E) of both patients and caregivers on quality of life of caregivers (O)?”.

### 2.1. Search Strategy

PubMed/Medline, Scopus, and Web of Science were systematically searched for articles published from the inception date of the databases to 4 August 2024. The following terms, along with Boolean operators (AND/OR), were used in the advanced search: (“caregiver” OR “carer” OR “family” OR “relative”) AND (“schizo*” OR “psychosis”) AND (“quality of life” OR “life satisfaction”). The full search string for each database is available in the Supplementary Materials.

### 2.2. Selection Process, Eligibility Criteria and Data Extraction

We used the Rayyan tool to automatically identify duplicates (Ouzzani et al., 2016). The duplicates were then resolved manually upon verification by one of the investigators (A.D.V or A.C.). Two independent reviewers (A.D.V. and A.C) performed title and abstract screening to identify articles potentially meeting the inclusion and exclusion criteria. After this step, the selected articles were sought for retrieval in full text and assessed against the inclusion and exclusion criteria. Any disagreement during title and abstract screening or full-text review was resolved either by consensus or by consulting with a senior author (V.D.).

The inclusion criteria used were as follows: (1) original, peer-reviewed articles assessing QoL in informal caregivers of PwS; (2) studies conducted on informal caregivers of adult PwS (age ≥ 18 years old); (3) the informal caregiver is an unpaid person (family member, close relative, partner, friend, neighbor, etc.) who provided regular care or assistance to a person diagnosed with schizophrenia; (4) studies investigated the relationship between the QoL of informal caregivers and any sociodemographic, clinical, or psychological variables of informal caregivers or patients; (5) studies reporting a quantitative analysis; (6) QoL was measured using any valid and reliable instrument designed to measure QoL; (7) the articles were published in English.

We excluded articles based on the following criteria: (1) qualitative research, literature reviews, case reports, letters to editors, abstracts presented at scientific meetings, book chapters, dissertations, and editorials; (2) studies conducted on caregivers of pediatric patients (age < 18 years old); (3) articles written in other languages than English; (4) studies conducted on caregivers of patients with any other mental disorder besides schizophrenia (e.g., bipolar disorder, psychosis, etc.); (5) studies using a specific scale of QoL (oral health QoL, sexual QoL, etc.); (6) studies conducted on caregivers who were paid to provide care to the patients.

Two researchers (A.C. and A.D.V.) independently extracted relevant data from the included articles in a customized Microsoft Excel spreadsheet. We sought data on the outcome of interest for this systematic review, namely any sociodemographic, clinical, or psychological variables of caregivers or patients that were investigated in relation to their impact on caregivers’ QoL, as quantified by any validated scale. Additionally, we extracted, in a summarized manner, the following data: publication characteristics (authors, year of publication, and country where the study was conducted), study characteristics (study design, setting of the patients, and sample characteristics—number of participants and percentage of female participants included, mean and standard deviation age), and QoL measure.

### 2.3. Methodological Quality Appraisal

The risk of bias was evaluated using the appropriate JBI checklist (i.e., for cross-sectional studies) (Moola et al., 2020). To obtain a final score, we used the method proposed by Černe Kolarič et al. (2024). More precisely, each “yes” answer received one point while no points were given for “no” and “unclear” answers. For each study, the total score was determined by summing the points from each question. The total score could range from 0 to 8 points. Percentages were calculated as well. We stratified the studies into four levels of methodological quality: low (<60%), moderate (60–79%), high (80–89%), and excellent (90–100%). All the studies, regardless of their methodological quality, were included.

Of note, no formal certainty of evidence assessment (i.e., GRADE) was conducted as the design of all the included studies was cross-sectional without comparison groups or effect estimates suitable for GRADE assessment (Schünemann et al., 2013). Also, due to data heterogeneity, we were not able to perform a meta-analysis. No sensitivity analysis was conducted as no meta-analysis was performed.

## 3. Results

### 3.1. Search Results

**Figure 1:**
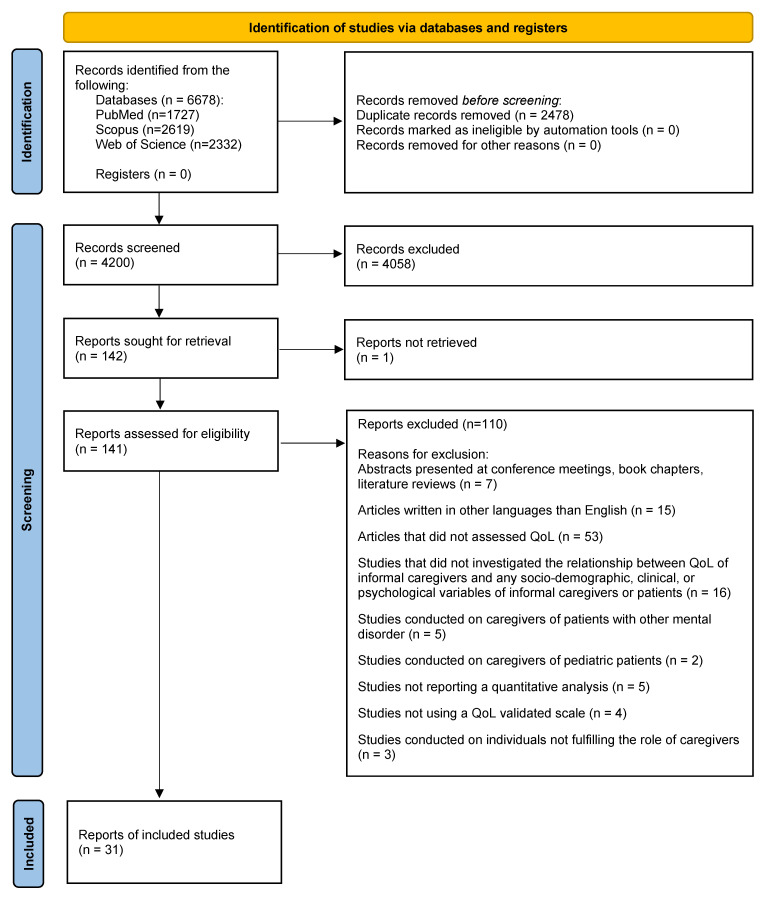
PRISMA flowchart of the study selection process (Page et al., 2021).

A total of 6678 records were identified after searching the three databases and 2478 records were detected as duplicates and removed. Out of the remaining 4200 records, 142 papers were sought for retrieval after the title and abstract screening step. Despite extensive efforts, including contacting the corresponding author and the journal’s editor-in-chief and searching multiple databases, the full text of one paper remained unavailable and was, thus, not included in the final analysis. One hundred and ten articles were excluded after a full-text assessment based on the inclusion and exclusion criteria (see Supplementary Materials—Table S2). In the end, 31 papers were included in this systematic review after completing all the methodological steps. A flowchart illustrating the selection process for articles included in this review is depicted in Figure 1.

### 3.2. Characteristics of the Included Studies

The studies included in this review were published between the years 2007 and 2024 (70.9% published in the last 10 years) and conducted on individuals from 19 countries. Most of the studies were conducted in India (*n* = 7), followed by China (*n* = 4). All the studies employed a cross-sectional design. WHOQoL-Bref was the most used QoL instrument (*n* = 20), followed by S-CGQoL (*n* = 7). Eighteen studies (*n* = 18) were conducted on caregivers of outpatients (out of 26 studies that reported the care setting). In most of the included investigations (*n* = 20), women accounted for over 50% of the caregiver samples. Detailed characteristics of each included study are presented in Table 1.

### 3.3. Critical Assessment and Methodological Quality of the Included Articles

The methodological quality of the studies included in this review ranges from moderate to excellent. However, an important number of studies (n = 14) were categorized as having high or excellent methodological quality. The most frequent source of risk of bias was the lack of identification of confounding factors. A synthesis of the methodological quality assessment using the JBI Critical Appraisal Checklist for Analytical Cross-Sectional Studies is presented in Table 2.

### 3.4. Sociodemographic Factors

#### 3.4.1. Gender

Out of the 31 studies, 13 examined the correlation between caregivers’ gender and their quality of life (QoL). Among these, only ten (Caqueo-Urízar et al., 2016a, 2016b; Marutani et al., 2020; Opoku-Boateng et al., 2017; Ribé et al., 2018; Stanley et al., 2017; Tristiana et al., 2019; Vadher et al., 2020; Wang et al., 2023; ZamZam et al., 2011) identified a statistically significant association, consistently indicating that male caregivers reported a higher QoL compared to female caregivers. In the remaining three studies, no statistically significant correlation was found.

#### 3.4.2. Age

Regarding the age of caregivers, 12 studies (Boyer et al., 2012; Caqueo-Urízar et al., 2016a; Francisquini et al., 2020; Hsiao et al., 2020; Khatimah et al., 2022; Li et al., 2007; Margetić et al., 2013; Peng et al., 2024; Ribé et al., 2018; Ukpong & Ibigbami, 2021; Usman et al., 2021; Yerriah et al., 2022) identified a negative correlation between increasing age and caregivers’ QoL, indicating that older caregivers tend to experience a decline in QoL. Conversely, only one study (Mizuno et al., 2012) reported a positive correlation, suggesting that aging may, in some cases, be associated with an improved QoL for caregivers.

#### 3.4.3. Educational Level

In 9 studies out of 31, the researchers assessed the relationship between caregivers’ education levels and their QoL. Among these, seven studies (Hsiao et al., 2020; Li et al., 2007; Opoku-Boateng et al., 2017; Peng et al., 2024; Ribé et al., 2018; Yerriah et al., 2022; ZamZam et al., 2011) found that a higher level of education positively influenced QoL. However, Khatimah et al. (2022) reported a negative correlation, suggesting that higher education predicted lower overall QoL. Meanwhile, Khan and Ghourmulla Alghamdi (2020) highlighted the role of caregiving knowledge—closely associated with education—as a factor that enhances QoL.

#### 3.4.4. Employment Status

Across 10 studies (Boyer et al., 2012; Caqueo-Urízar et al., 2016a; Hsiao et al., 2020; Marutani et al., 2020; Ribé et al., 2018; Ukpong & Ibigbami, 2021; Usman et al., 2021; Winahyu et al., 2015; ZamZam et al., 2011), employment was identified as a significant sociodemographic factor influencing caregivers’ QoL. Consequently, caregivers who are employed tend to report a higher quality of life compared to those who are not.

#### 3.4.5. Kinship

Regarding kinship, six studies examined the association between kinship and caregivers’ QoL, yielding heterogeneous findings. Three studies (Hsiao et al., 2020; Margetić et al., 2013; ZamZam et al., 2011) reported that being a parent of an individual with schizophrenia was associated with lower QoL, while two studies (Boyer et al., 2012; Caqueo-Urízar et al., 2016a) specifically indicated that mothers exhibited lower QoL scores. Conversely, Usman et al. (2021) found that parental caregiving had a positive impact on QoL.

#### 3.4.6. Living Situation

Only four studies investigated the correlation between caregivers’ living situation and their QoL. Three of them (Boyer et al., 2012; Mizuno et al., 2012; Ribé et al., 2018) indicated that residing with the patient negatively impacts caregivers’ QoL. Additionally, Geriani (2015) reported that caregivers from nuclear families exhibited better QoL in the environmental domain compared to those from joint families.

#### 3.4.7. Marital Status

Compared to being single, being married has been shown to be related to better QoL in four studies (Margetić et al., 2013; Opoku-Boateng et al., 2017; Tristiana et al., 2019; Yerriah et al., 2022). In contrast, Ukpong and Ibigbami (2021) indicated that married caregivers had lower QoL in the psychological health, social relationships, and environmental domains.

#### 3.4.8. Family Income

Multiple studies (Caqueo-Urízar et al., 2016b; Hsiao et al., 2020; Li et al., 2007; Marutani et al., 2020; Peng et al., 2024; Stanley et al., 2017; Usman et al., 2021; Yerriah et al., 2022) established a direct correlation between caregivers’ economic status and their QoL, indicating that financial stability enhances QoL, while financial strain significantly diminishes it.

#### 3.4.9. Physical Health

Regarding the physical health of caregivers, four studies (Li et al., 2007; Marutani et al., 2020; Usman et al., 2021; ZamZam et al., 2011) highlighted that the presence of physical illness among caregivers was significantly associated with a lower quality of life in the physical, psychological, and environmental domains.

#### 3.4.10. Time Spent Caregiving

A limited number of studies have identified a significant inverse relationship between caregiving hours and quality of life. Ukpong and Ibigbami (2021) suggested that the duration of caregiving is a key determinant of reduced QoL across the physical, psychological, and social domains. Quah (2014) reported that caregivers who provided more than six hours of daily care (role overload) exhibited a lower QoL. Furthermore, ZamZam et al. (2011) reported that the caregivers of patients enrolled in day care programs experienced lower QoL across all the domains.

#### 3.4.11. Ethnicity

In two different studies (Caqueo-Urízar et al., 2016a, 2016b), it was indicated that Aymara ethnicity determined lower QoL scores in the relationships with family and material burden dimensions. These studies were conducted in three Latin American cities—La Paz (Bolivia), Arica (Chile), and Tacna (Peru)—and specifically examined the experiences of both Aymara (an Indigenous ethnic group native to the Andes region) and non-Aymara individuals. It was also noted that caregivers belonging to an ethnic minority, specifically the Aymara, reported lower QoL overall.

#### 3.4.12. Patients’ Sociodemographic Factors

Regarding patients’ sociodemographic factors, Ukpong and Ibigbami (2021) reported that an increase in patient age is associated with a decline in caregivers’ QoL in the physical domain. In contrast, Usman et al. (2021) found that the caregivers of patients older than 30 years had a higher QoL compared to those caring for younger patients. Moreover, ZamZam et al. (2011) suggested that the caregivers of employed patients or those with higher educational attainment exhibit an enhanced QoL in the psychological domain. Additionally, Opoku-Boateng et al. (2017) reported that closer proximity to psychiatric services is positively correlated with an overall improvement in caregivers’ QoL.

### 3.5. Patient’s Clinical Factors

#### 3.5.1. Duration of Illness

Three studies (Li et al., 2007; Ukpong & Ibigbami, 2021; ZamZam et al., 2011) indicated that a longer duration of the patient’s illness is associated with a decline in the caregiver’s QoL. Furthermore, an earlier onset of schizophrenia appears to contribute to a further reduction in the caregiver’s QoL (Caqueo-Urízar et al., 2016a; Ribé et al., 2018; ZamZam et al., 2011).

#### 3.5.2. Symptom Severity

Among the 31 studies, 6 (Caqueo-Urízar et al., 2016a; Hsiao et al., 2020; Peng et al., 2024; Stanley et al., 2017; ZamZam et al., 2011) reported a decline in caregivers’ QoL as the severity of patients’ symptoms increased. Additionally, regarding patients’ functionality level, Khatimah et al. (2022) examined the correlation with caregivers’ QoL, concluding that higher patient functionality is associated with an improved QoL for caregivers.

#### 3.5.3. Number of Hospitalizations and Comorbidities

Several studies (Li et al., 2007; ZamZam et al., 2011) have identified a negative correlation between the number of hospitalizations of PwS and the QoL of their caregivers. As for patients with comorbidities, caregivers experience a lower quality of life, particularly in the physical domain, as a result of the more rapid and severe deterioration of the patient’s health.

### 3.6. Caregiver’s Psychological Factors

#### 3.6.1. Burden of Care

The most commonly observed association was between caregiving burden and caregivers’ QoL, with 15 out of the 31 studies reporting this correlation, consistently indicating a negative relationship (Khan & Ghourmulla Alghamdi, 2020; Caqueo-Urízar et al., 2016b; Geriani, 2015; Ghosh et al., 2020; Kate et al., 2013b; Opoku-Boateng et al., 2017; Quah, 2014; Ribé et al., 2018; Stanley et al., 2017; Tristiana et al., 2019; Ukpong & Ibigbami, 2021; Vadher et al., 2020; Wang et al., 2023; Winahyu et al., 2015; Yerriah et al., 2022). Quah (2014) highlights a strong association between role distress, resulting from caregiving overload, and a decline in QoL.

#### 3.6.2. Coping Strategies

Regarding coping strategies, four articles presented different perspectives on their impact on quality of life (QoL). Hsiao et al. (2020) highlighted that effective family coping strategies were positively associated with improved QoL across all the domains. Kate et al. (2014) identified a negative correlation between collusion and QoL, particularly in the domains of physical health, social relationships, and environment, while coercion was negatively correlated with general health and environmental QoL. Geriani (2015) reported that effective coping strategies were linked to an enhancement in environmental QoL. Finally, Mizuno et al. (2012) discussed the concept of a sense of coherence, which was positively correlated with overall QoL.

#### 3.6.3. Depression, Anxiety, and Stress

Khatimah et al. (2022) and Opoku-Boateng et al. (2017) identified depression, anxiety, and stress as significant contributors to a decline in caregivers’ QoL. Similarly, Ukpong and Ibigbami (2021) reported that anxiety and depressive symptoms are strongly associated with lower QoL. Furthermore, Stanley and Balakrishnan (2023) demonstrated that stress exhibited significant direct effects on both resilience and QoL, indicating a substantial impact on these variables. Furthermore, resilience functioned as a mediator in the relationship between stress and QoL, with a significant indirect effect, suggesting that higher resilience partially attenuates the adverse influence of stress on QoL. ZamZam et al. (2011) highlighted that an increase in caregiver stress levels is associated with a decline in QoL, particularly within the psychological and environmental domains.

#### 3.6.4. Social and Family Support

Opoku-Boateng et al. (2017) identified a positive correlation between family support and caregivers’ QoL. Kate et al. (2014) reported a negative correlation between seeking social support and QoL. In contrast, the studies conducted by Ribé et al. (2018), Stanley and Balakrishnan (2023), and Winahyu et al. (2015) demonstrated that strong social support positively influenced the psychological, social, and environmental QoL domains.

#### 3.6.5. Hope and Religious Involvement

Francisquini et al. (2020) identified a positive correlation between hope and quality of life (QoL). Conversely, Caqueo-Urízar et al. (2016a) reported that while religious involvement was not significantly associated with overall QoL, it exhibited a negative correlation, specifically within the psychological and physical well-being domains. However, Kate et al. (2013a) found that higher scores in spirituality-related beliefs were associated with improved QoL, and similarly, greater levels of spirituality, religiousness, and personal beliefs correlated with enhanced QoL.

#### 3.6.6. Affiliate Stigma and Expressed Emotion

Wang et al. (2023) and Peng et al. (2024) found a significant negative correlation between affiliate stigma and QoL, indicating that higher levels of affiliate stigma were associated with lower QoL among caregivers. Additionally, Peng et al. (2024) highlighted that expressed emotion significantly diminishes the QoL of caregivers.

## 4. Discussion

The main contribution of this systematic review was to highlight the key influencing factors of the QoL of caregivers of PwS. We observed that the QoL of caregivers for PwS is influenced by a complex interplay of sociodemographic, clinical, and psychological factors, with caregiving burden and mental health challenges emerging as particularly critical determinants. According to our search results, interest in researching the factors influencing the QoL of caregivers of PwS emerged in 2007 and has grown significantly over the past 5–6 years. Considering the numerous factors related to QoL, this section will focus on those that exhibit a stronger degree of association.

Based on the frequency and consistency of their impact across studies, the most important sociodemographic factors we found to influence caregivers’ QoL are likely to be gender, age, employment status, education level, and family income with male, younger, employed caregivers, and those with higher education and family income generally reporting better QoL.

The results presented herein show that caregivers’ age demonstrates an inverse relationship with their QoL. Specifically, as caregivers age, a decline in their QoL is observed, likely attributable to the deterioration of their own health and the onset of multiple comorbidities, which concurrently impair their capacity to effectively perform caregiving duties (Alcañiz & Solé-Auró, 2018; Gabriel & Bowling, 2004; Lima-Rodríguez et al., 2022). One study (Mizuno et al., 2012) observed that aging was associated with an increase in caregivers’ QoL, a finding echoed by a limited number of similar studies (Neikrug, 2003; Volanen et al., 2004). This outcome can be explained by the notion that elderly caregivers, through accumulated experience, develop a deeper understanding and recognition of their patients, and they gain insight into their own limitations and build confidence in their coping strategies.

Regarding gender, all the studies concluded that female caregivers, particularly mothers, reported a lower QoL compared to their male counterparts. This disparity can be attributed to multiple factors, including differences in coping strategies, insufficient social support systems that address their specific needs, and sociocultural expectations that place caregiving responsibilities on women regardless of personal circumstances and challenges (Vadher et al., 2020). These factors compound the difficulty of balancing caregiving duties with other socially prescribed domestic roles (Adewuya et al., 2011).

Most of the reviewed studies indicate that a higher level of education tends to provide caregivers with improved problem-solving abilities and coping mechanisms, enabling them to manage caregiving responsibilities more effectively (ZamZam et al., 2011). Moreover, it facilitates their ability to navigate various life challenges, engage in leisure activities, pursue hobbies that improve the quality of their free time, and access cultural events. Additionally, education provides opportunities for social interaction with individuals who share similar intellectual and artistic interests, further enriching their personal and social well-being. Furthermore, those with higher education levels are more likely to secure stable, well-compensated employment, which helps reduce the financial burdens associated with caregiving (Awadalla et al., 2005; ZamZam et al., 2011). Only one study (Khatimah et al., 2022) has suggested that there is a negative correlation between the level of education and the overall QoL of caregivers. This finding may be explained by the increased awareness of the challenges that caregivers face when caring for PwS. Such awareness often leads to heightened stress levels, particularly due to the chronic nature and progression of the disease. Furthermore, caregivers with higher education levels may have more ambitious professional goals or demanding career responsibilities. These professional commitments require significant time and energy, which may create a sense of imbalance and dissatisfaction with the caregiving role, potentially contributing to a decline in their overall QoL.

The employment status of caregivers is consistently associated in the studies we reviewed with improved overall QoL. One possible explanation for this relationship is the enhanced financial stability that employment provides, which facilitates access to necessary resources to meet caregiving demands (Hsiao et al., 2020; Lima-Rodríguez et al., 2022). Beyond the financial advantages, employment offers caregivers a valuable opportunity for respite, allowing them a break from the demands of caregiving and enhancing their emotional and psychological well-being. The combined effects of financial stability and the mental health benefits of employment are likely key factors in improving caregivers’ overall QoL (ZamZam et al., 2011). Additionally, employed caregivers often benefit from a broader social network, which can offer emotional and practical support (Hsiao et al., 2020; Leng et al., 2019). Employment is also linked to higher self-esteem and greater satisfaction with services, both of which contribute positively to caregivers’ QoL (Gold et al., 2016; Lima-Rodríguez et al., 2022).

For the same reason, in accordance with employment status, family income is positively correlated with the QoL of caregivers of PwS in all the studies that considered this factor. This is likely due to, as previously mentioned, the enhanced access to various material resources and services necessary for these patients, which are facilitated by a stable and substantial financial income. Additionally, caregivers with higher incomes and more stable careers may be able to afford external assistance with daily tasks—such as hiring domestic help or accessing respite care—which can substantially reduce the physical and emotional burden of caregiving. For instance, in Spain, receiving a caregiving subsidy or home care support was associated with an increase in satisfaction with life by approximately 15% and 10.5%, respectively (Costa-Font & Vilaplana-Prieto, 2022).

Sociodemographic factors related to the patients have been studied less extensively. With regard to patient age, the findings we found have been inconsistent. One study (Ukpong & Ibigbami, 2021) indicated that as patients age, there is a corresponding decline in the caregiver’s quality of life. This may be due to the fact that as patients grow older, the progression of their illness tends to accelerate, resulting in increased caregiving demands and a significantly higher time commitment. In contrast, another study (Usman et al., 2021) found that the caregivers of patients over the age of 30 reported a higher QoL. This could be explained by the fact that a longer duration since diagnosis is associated with reduced perceived burden, increased personal resourcefulness, and improved mental health, suggesting that people may gradually adapt to the caregiving role over time when caring for a family member with a severe mental illness (Lima-Rodríguez et al., 2022; Zauszniewski et al., 2008).

Regarding the educational level and employment status of the patient, one study found that both factors are positively correlated with the QoL of caregivers. This can be attributed to the fact that higher educational attainment and employment are likely to promote greater independence for the patient, reduce the financial burden on the caregiver, and enhance communication skills, all of which contribute to a more effective and harmonious caregiver–patient relationship (Lima-Rodríguez et al., 2022; ZamZam et al., 2011).

In terms of clinical factors influencing caregivers’ QoL, the most impactful factor is symptom severity, as it has a direct, substantial effect on all the domains of QoL, including physical, psychological, and emotional well-being. However, all the clinical factors examined were associated with the progression of the disease, which in turn leads to a decline in the QoL of caregivers.

A limited number of studies have suggested that caregiving for a patient with an earlier onset may contribute to a deterioration in caregivers’ QoL (ZamZam et al., 2011). This may be explained by the severe cognitive impairments commonly observed in patients with youth-onset schizophrenia. These individuals tend to exhibit more pronounced deficits in areas such as arithmetic, executive function, IQ, psychomotor processing speed, and verbal memory compared to those with first-episode schizophrenia. In contrast, patients with late-onset schizophrenia show fewer deficits in arithmetic, digit-symbol coding, and vocabulary, but experience more significant impairments in attention, fluency, global cognition, IQ, and visuospatial construction (Rajji et al., 2009).

In terms of symptom severity, it is well established that the intense positive symptoms of schizophrenia, including hallucinations, aggression, and destructive behavior, lead to considerable distress for caregivers (Schulz & Sherwood, 2008; ZamZam et al., 2011). It has been proposed that the acute, although episodic, nature of positive symptoms makes them more challenging for caregivers to manage, in contrast to the more persistent but less immediately threatening negative symptoms. This disparity in symptom characteristics contributes to the heightened burden faced by caregivers (Dyck et al., 1999; Schulz & Sherwood, 2008; ZamZam et al., 2011).

The primary psychological factors found by this review to affect caregivers’ QoL are caregiving burden, mental health challenges (such as depression, anxiety, and stress), coping strategies, and social support. Notably, caregiving burden and mental health issues exert the most substantial negative impact on caregivers’ overall QoL.

The caregiving burden for individuals caring for PwS is multifaceted, affecting caregivers’ physical, emotional, psychological, and social well-being. The key consequences of this burden include disruption in family life and interactions, financial strain, and adverse impacts on overall well-being and health (Stanley et al., 2017; Talwar & Matheiken, 2010). To effectively address these challenges, comprehensive support systems are essential. These should include financial assistance, access to healthcare resources, and emotional support to alleviate the strain on caregivers and improve their quality of life (Opoku-Boateng et al., 2017).

Mental health issues such as depression, anxiety, and stress are significant factors contributing to the decline in caregivers’ QoL, a finding supported by a considerable body of research. Khatimah et al. (2022) and Opoku-Boateng et al. (2017) highlight the negative impact of these mental health challenges on caregivers’ QoL, with Ukpong and Ibigbami (2021) further corroborating this by identifying a strong association between anxiety and depressive symptoms and lower QoL. These findings suggest that mental health challenges severely decrease caregivers’ ability to manage their caregiving responsibilities, leading to increased distress and diminished life satisfaction.

Stanley and Balakrishnan (2023) introduced an additional perspective by showing how stress directly affects both resilience and QoL. The research suggests that resilience can mediate the impact of stress, meaning that caregivers with higher resilience may experience less of a decline in QoL, even in the face of stress. This underscores the importance of fostering resilience as a strategy to mitigate the detrimental effects of caregiving-related stress. Finally, ZamZam et al. (2011) reinforced these findings by demonstrating that higher levels of caregiver stress are closely linked to a decrease in QoL, especially within the psychological and environmental domains. This highlights the complex nature of caregiver stress and its wide-ranging effects on multiple aspects of their lives.

The findings of this review have several implications for practice, policy, and future research. Together with data highlighting lower QoL for caregivers of PwS as compared to controls (Cohen et al., 2022; Hayes et al., 2015), our findings indicate the immediate need for interventions aimed at alleviating burden and improving QoL. Usually, most current interventions focus on providing caregivers with information about the illness and treatment, as well as education on the management of stress, self-care and well-being, strategies for handling patients’ symptoms or challenging behaviors, etc. Through an RCT, Martín-Carrasco et al. (2016) proved that a psychoeducational interventional program consisting of 12 in-person sessions reduced burden and depressive symptoms at the end of the intervention and 4 months later (Martín-Carrasco et al., 2016). Hasan et al. (2015) conducted an RCT to assess the impact on burden and QoL of an intervention delivered through six psychoeducational booklets. Post-intervention and 3 months later, the participants allocated to the psychoeducational program had lower burden and better QoL as compared to the control group (Hasan et al., 2015). In addition, a recent meta-analysis encompassing 439 caregivers from five studies showed that psychoeducation has a beneficial effect on QoL (Chow et al., 2024). Although online interventions might have the advantage of being more accessible and cost-efficient, they have yielded mixed outcomes for caregivers of PwS. Nevertheless, a relatively recent systematic review concludes that they might represent a promising therapeutic approach (Barbeito et al., 2020). The results from this review could contribute to tailoring such interventions. More precisely, interventions should specifically focus on the most vulnerable caregiver group, including unemployed older females with lower educational attainment and facing financial difficulties. In light of the findings from this review, we recommend that interventions be initiated in the early stages of the illness, ideally around diagnosis, to provide caregivers with timely support and knowledge, thereby helping to prevent the deterioration of their quality of life as the illness progresses. While the optimal duration of the intervention remains unclear, sustained support over time, with periodic follow-ups, would be optimal considering the chronic and relapsing nature of schizophrenia. A multidisciplinary team (i.e., psychiatrist, psychologist/psychotherapist, psychiatric nurse, social worker, legal counsel, etc.) would be best suited to address the complex range of challenges. Additionally, welfare policies should be designed and implemented to support this at-risk group of caregivers of PwS. The results that emerged from this systematic review suggest that routine screening for burden, anxiety, depression, and psychological distress symptoms should be incorporated into everyday practice for early detection. Psychoeducational interventions should include measures for preventing and/or reducing such symptoms to maintain or improve QoL. Finally, since the patient’s clinical factors impact the caregiver’s QoL also, continuous efforts should be made to reduce the duration of untreated psychosis, the number of hospitalizations, and the symptoms’ severity while improving community services and implementing multidisciplinary approaches. Longitudinal assessment of the factors influencing QoL has emerged as a gap in the current research and is worth undertaking in future studies.

This review has several limitations that should be noted. Firstly, it included studies published only in English; therefore, other relevant studies in other languages may not have been taken into discussion. Furthermore, an additional limitation is the inability to access the full text of one study despite our extensive efforts to obtain it. Thirdly, publication bias, particularly the tendency not to publish studies with negative results, may have limited our ability to provide a comprehensive and detailed overview of the factors influencing QoL in caregivers of PwS. Fourthly, most of the studies included in this review used small population samples and failed to identify confounding factors (according to our methodological appraisal). Therefore, the absence of multivariate analysis in several studies, which reduces the ability to control for confounding variables, undermines the strength and generalizability of the reported associations. This limitation underscores the need for future research with more rigorous designs and appropriate statistical analysis. All the studies used a cross-sectional design; therefore, we could not describe which factors are involved in shaping the longitudinal evolution of QoL. In addition, QoL measurement inconsistency is another limitation. Studies using different QoL scales (some population-specific, others generic) may capture different aspects of QoL, leading to a certain variability in the reported outcomes. This systematic review does not include a formal certainty of evidence assessment (i.e., GRADE) as GRADE is intended to assess the certainty of evidence for specific outcomes based on comparative effect estimates. As such, it might not be suitable for descriptive cross-sectional studies without comparative groups or effect measures (Schünemann et al., 2013). Therefore, the level of certainty of evidence needs to be interpreted with caution. Another limitation is the inability to perform a meta-analysis due to the heterogeneity of data. Lastly, since the included studies originate from specific geographical regions (note most studies came from India and China), the results lack generalizability to other populations.

## 5. Conclusions

Our review highlights certain sociodemographic, clinical, and psychological factors that influence the QoL of caregivers of PwS. The findings indicate that younger male caregivers and those employed, with higher education and higher family income, generally reported better QoL. Furthermore, some of the data indicate that schizophrenia symptom severity may also influence caregivers’ QoL. Psychological factors, particularly depressive and anxiety symptoms, and caregiver burden may also play an important role in determining QoL. Resilience, internalized stigma, coping strategies, and social support have been linked to having an impact on QoL, but further studies are needed to support these results. While these findings are promising, future research is warranted and should focus on longitudinal studies to better understand which factors influence the dynamic nature of QoL in caregivers of PwS.

Targeted interventions aimed at supporting caregivers, such as psychoeducation-based interventions, were shown to reduce perceived burden and improve quality of life (Chow et al., 2024; Di Lorenzo et al., 2024). By mapping the factors influencing QoL, this review further reinforces the urgent need to increase the availability of these interventions by effectively integrating them into mental health services.

## Figures and Tables

**Table 1:** Characteristics and key findings of the included studies.

Author and Year	Country Study Design *n*, Mean (SD) Age, % of Female Caregivers Patients’ Setting	QoL Measure	Main Findings		
			Sociodemographic Factors of Caregivers	Patient’s Sociodemographic and Clinical Factors	Psychological Factors of Caregivers
(Ribé et al., 2018)	Spain Cross-sectional *n* = 100, 60.1 (11.7), 82% Outpatients	WHOQoL-Bref	⇑ age—⇓ QoL (physical and social domains). ♀—⇓ QoL (physical and social domains). ⇑ education—⇑ QoL (social and environmental domains). Employment—⇑ QoL (physical domain). Living with the patient—⇓ QoL (social and environmental domains).	Early-onset schizophrenia in patients—⇓ QoL	⇑ caregiving burden—⇓ QoL in all domains. Strong social support—⇑ psychological, social, and environmental QoL domains. ⇑ Family APGAR scores—⇑ QoL.
(Stanley et al., 2017)	India Cross-sectional *n* = 75, 49.4, 37.5% Inpatients	S-CGQoL	No significant correlation was found between caregiver age and QoL. ♂—⇑ QoL (mean = 93.9) than ♀ (mean = 91.2). ⇓ income—⇓ QoL.	⇑ severe symptoms—⇓ QoL (*p* < 0.05).	⇑ caregiver burden—⇓ QoL across all domains (r = −0.34, *p* < 0.01).
(Kate et al., 2014)	India Cross-sectional *n* = 100, 46 (11.6), 35% Outpatients and inpatients	WHOQoL-Bref	Rural background—⇑ QoL in the social health domain.	Clinical variables of the patients, including symptom severity and illness duration, showed no significant correlations with caregivers’ QoL.	Coping styles: seeking social support—⇓ QoL; collusion—⇓ QoL (physical health, social relationships, and environmental domains); and coercion—⇓ QoL (general health and environmental domains). ⇑ spirituality, religiousness, and personal beliefs—⇑ QoL.
(Margetić et al., 2013)	Croatia Case–control *n* = 138, 52.6 (14.4), 63.8% Outpatients	QLESQ-SF	⇑ age—⇓ QoL (r = −0.464, *p* = 0.0001). Parents and children of patients—⇓ QoL than siblings (F = 3.567, *p* = 0.031). Single, divorced, or widowed caregivers—⇓ QoL.	QoL—no correlation with PANSS scores, GAF scores, length of illness, or number of hospitalizations.	n.s.
(Mizuno et al., 2012)	Japan Cross-sectional *n* = 34, 63.3 (13.3), 79.4% Outpatients	WHOQoL-Bref	⇑ age—⇑ psychological and environment QoL scores. Living with patients—⇓ psychological and environmental QoL.	n.s.	⇑ sense of coherence—⇓ QoL.
(Quah, 2014)	Singapore Cross-sectional *n* = 47, no mean age, 55.3% Patients’ setting n.s.	WHOQoL-Bref	n.s.	n.s.	Caregivers providing more than 6 h daily (role overload)—⇓ QoL. ⇑ role distress—⇓ QoL across all domains.
(Kate et al., 2013b)	India Cross-sectional *n* = 100, 45.9 (11.6), 35% Outpatients and inpatients	WHOQoL-Bref	n.s.	n.s.	⇑ caregiver burden, especially in the tension domain—⇓ QoL across all domains.
(Stanley & Balakrishnan, 2023)	India Cross-sectional *n* = 75, 46.24, 62.7% Inpatients	The Satisfaction with Life Scale	n.s.	n.s.	Stress had significant direct effects on resilience (effect = 1.39, *p* < 0.01) and QoL (effect = 0.70, *p* < 0.001). Resilience mediated the relationship between stress and QoL (effect = 0.09, *p* < 0.01). Social support did not moderate the relationship between stress and resilience (*p* > 0.05) but was (+) correlated with resilience and QoL.
(Khatimah et al., 2022)	Indonesia Cross-sectional *n* = 106, 43, 85.8% Outpatients	WHOQoL-Bref	⇑ age—⇓ QoL (in the physicological and environmental domains). Higher education—⇓ QoL (in the psychological and environmental domains (β = −0.775, *p* = 0.005)). Employed caregivers—⇑ QoL (in the environmental and psychological domains (β = 1.869, *p* = 0.008, β = 1.112, *p* = 0.035)).	General functioning of patients—⇑ QoL.	⇑ stress—⇓ QoL in all domains: physical health (β = −0.134, *p* = 0.004); environment (β = −0.134, *p* = 0.004); and overall QoL (β = −0.224, *p* = 0.033). Anxiety and depression (−) impacted QoL scores. Environment: depression (β = −0.156, *p* = 0.005); anxiety (β = −0.149, *p* = 0.001). Social relationships: depression (β = −0.068, *p* = 0.012); overall QOL (β = −0.232, *p* = 0.023; β = −0.335, *p* = 0.004)
(Tristiana et al., 2019)	Indonesia Cross-sectional *n* = 222, 44.05 (9.61), 31.1% Outpatients	S-CGQoL	♂—⇑ QoL (*p* = 0.003). Married caregivers—⇑ QoL (*p* = 0.045). Longer caregiving duration—no significant correlation with QoL.	n.s.	⇑ caregiving burden—⇓ QoL (r = −0.434, *p* < 0.001).
(Francisquini et al., 2020)	Brazil Cross-sectional *n* = 117, 56.7 (15.7), 74.4% Outpatients	WHOQoL-Bref	⇑ age—⇓ QoL, particularly in social relationships (r = −0.401, *p* < 0.01).	n.s.	⇑ hope—⇓ QoL in all domains (r = 0.342–0.595, *p* < 0.05).
(Caqueo-Urízar et al., 2016b)	Bolivia, Chile, Peru Cross-sectional *n* = 253, 54.7 (14.4), 67.7% Outpatients	S-CGQoL	n.s.	⇑ neurocognitive deficits—⇓ QoL (r = −0.32, *p* < 0.05).	n.s.
(Caqueo-Urízar et al., 2016a)	Bolivia, Chile, Peru Cross-sectional *n* = 253, 54.7 (14.4), 67.7% Outpatients	S-CGQoL	♀—⇓ QoL in the material burden dimension (β = −0.15, *p* < 0.05). Mothers—⇓ QoL in the psychological and physical well-being and psychological burden and daily life domains. Aymara ethnicity—⇓ QoL scores in the relationships with family (β = −0.17, *p* < 0.05) and material burden (β = −0.24, *p* < 0.01) dimensions. ⇑ income—⇑ QoL in the material burden domain (β = 0.25, *p* < 0.01).	⇑ severe symptoms—⇓ QoL in psychological and physical well-being (β = −0.29, *p* < 0.01) and psychological burden and daily life (β = −0.28, *p* < 0.01). Earlier onset of schizophrenia—⇓ QoL. Unawareness of mental disorder—⇓ QoL (β = −0.23, *p* < 0.001). ⇑ patients age—⇑ QoL in the material burden dimension (β = 0.19, *p* < 0.05).	⇑ subjective burden—⇓ QoL (in psychological and physical well-being). Religious Involvement—not significantly associated with overall QoL, except for a (-) correlation in the psychological and physical well-being domains (β = −0.17, *p* < 0.05).
(Khan & Ghourmulla Alghamdi, 2020)	Saudi Arabia Cross-sectional *n* = 150, 44.7 (13.1), 65.3% Patients’ setting n.s.	S-CGQoL	There is no significant difference between gender means. Higher knowledge about schizophrenia—⇑ QoL (r = 0.236, *p* < 0.01). The interaction between knowledge and gender significantly affected QoL (three-way interaction R^2^ change = 3.64%, F = 7.24, *p* < 0.01).	n.s.	⇑ caregiving burden—⇓ QoL (r = −0.43, *p* < 0.01).
(Geriani, 2015)	India Cross-sectional *n* = 110, 43.82, 73% Patients’ setting n.s.	WHOQoL-Bref	Caregivers from nuclear families—⇑ environmental QoL compared to those from joint families (*p* < 0.01).	n.s.	⇑ burden levels—⇓ QoL in the physical, psychological, social, and environmental domains (*p* < 0.05). Good coping strategies—⇑ environmental QoL (*p* = 0.037).
(Marutani et al., 2020)	Cambodia Cross-sectional *n* = 59 Outpatients	SF-12 v1	♂—⇓ QoL (mental health component). ⇓ household economic status (insufficient to meet basic needs)—⇓ QoL. Employed caregivers—⇑ QoL. Caregivers with chronic physical disease—⇓ QoL (physical component).	The severity of the patient’s illness, the caregiving duration, and DUP—no direct correlation with the caregiver’s QoL.	n.s.
(Ukpong & Ibigbami, 2021)	Nigeria Cross-sectional *n* = 100, 56.13 (12.99), 63% Outpatients	WHOQoL-Bref	⇑ age—⇓ QoL (in all domains). Married caregivers—⇓ QoL in the psychological health, social relationships, and environmental domains. ⇑ caregiving duration—⇓ QoL (in the physical, psychological, and social domains.	⇑ illness duration—⇓ QoL (in the physical, psychological, and environmental domains). Employed patient—⇑ caregiver QoL in the physical domain. ⇑ patient’s age—⇓ QoL (physical domain).	⇑ caregiving burden—⇓ QoL across the psychological, social, and environmental domains. Depressive and anxiety symptoms—⇓ QoL in the psychological, social, and environmental domains.
(Wang et al., 2023)	China Cross-sectional *n* = 253, 47.43% Patients’ setting n.s.	WHOQoL-Bref	♂—⇑ QoL score (mean = 85.14, SD = 11.27) compared to ♀(mean = 82.35, SD = 10.09).	n.s.	⇑ levels of affiliate stigma—⇓ QoL. ⇑ caregiving burden—⇓ QoL (in all domains).
(Opoku-Boateng et al., 2017)	Ghana Cross-sectional *n* = 444, 47, 57% Outpatients	WHOQoL-Bref	♀—⇓ QoL. Caregivers with tertiary education—⇑⇑ QoL. Married caregivers—⇑ QoL. Close proximity to the psychiatric service—⇑ overall QoL. Receiving support from family in caregiving—⇑ physical and psychological QoL.	n.s.	Depression, anxiety, and stress levels—⇓ QoL. ⇑ caregiving burden—⇓ QoL.
(Yerriah et al., 2022)	South Africa Cross-sectional *n* = 101, 53.1 (14.2), 82.2% Outpatients and inpatients	WHOQoL-Bref	⇑ age—⇓ QoL (in the physical and social domains). ⇑ education levels—⇑ QoL (in the physical and social domains). Being married—⇑ QoL in the social domain score. ⇑ income—⇑ QoL in the physical, social, and environmental domains.	Patient with comorbidity—⇓ QoL physical domain.	⇑ caregiving burden—⇓ QoL (in all domains).
(Boyer et al., 2012)	France, Chile Cross-sectional French: n = 245, 60.6, 67.1% Chilean: *n* = 41, 54.3, 63.4% Outpatients	SF-36	Mothers—⇓⇓ QoL across all domains. Living with the patient—⇓ QoL mental composite domain. Employed caregivers—⇑ QoL in the physical composite domain. ⇑ age—⇓ QoL in the physical composite domain.	n.s.	n.s.
(Kate et al., 2013a)	India Cross-sectional *n* = 100, 46 (12), 35% Outpatients and inpatients	WHOQoL-Bref	No significant correlations were found between sociodemographic factors and caregiver QoL.	n.s.	⇑ positive caregiving experience—⇑ QoL in all domains. ⇑ scores in spirituality, religiousness, and personal beliefs—⇑ QoL.
(Li et al., 2007)	China Cross-sectional *n* = 96, 47 (12), 57.3% Inpatients	WHOQoL-Bref	⇑ age—⇓ QoL (physical health and psychological domains). ⇑ household income—⇑⇑ QoL (in the physical and psychological domains). ⇑ education levels—⇑ psychological, social, and environmental QoL. ⇑ caregiver physical health—the strongest predictor of ⇑ QoL.	⇑ illness duration and more hospitalizations of the patient—⇓ QoL.	n.s.
(ZamZam et al., 2011)	Malaysia Cross-sectional *n* = 117, 47 (12), 52.1% Outpatients	WHOQoL-Bref	Parents—⇓ QoL in the physical health domain. Higher education—⇑ QoL. Employed caregivers—⇑ QoL (in the physical and psychological domains). ♀—⇓ QoL in the physical domain. ⇑ caregiver health—⇑ QoL in the physical, psychological, and environmental domains.	⇑ illness duration and multiple hospitalizations—⇓ QoL. Later illness onset—⇑ social relationships QoL. Caregivers of patients attending day care programs—⇓ QoL across all domains. ⇑ severity of symptoms—⇓ QoL in the physical and environmental domains. Caregivers of employed patients or of patients with higher educational levels—⇑ QoL in the psychological domain.	⇑ caregiver stress levels—⇓ QoL in the psychological and environmental domains.
(Vadher et al., 2020)	India Cross-sectional *n* = 50, 46.7 (14.2), 40% Outpatients	WHOQoL-Bref	♀—⇓ QoL in the environmental domain.	n.s.	⇑ caregiver burden of care—⇓ QoL in all domains.
(Usman et al., 2021)	Pakistan Cross-sectional *n* = 150, 45.36 (3.85) 36.7% Outpatients	WHOQoL-Bref	Caregivers aged 31–60 years—⇑ QoL, compared to those aged < 30 years or > 60 years. Employed caregivers—⇑ QoL (*p* = 0.040). ⇑ household income—⇑⇑ QoL. Parents—⇑ QoL scores compared to spouse, offspring, sibling, or other. Caregivers with chronic physical illnesses—⇓⇓ QoL. Caregivers of patients aged >30 years—⇑ QoL compared to caregivers of younger patients (*p* < 0.001).	n.s.	n.s.
(Hsiao et al., 2020)	China Cross-sectional *n* = 157, 54.94 (2.22) 63.7% Inpatients	WHOQoL-Bref	⇑ age—⇓ QoL (in the physical and psychological domains). ⇓ education—⇓ QoL. ⇓ income—⇓ QoL in all domains. Employed caregivers—⇑ physical, psychological, and social QoL. Parents—⇓ QoL scores.	⇑ psychiatric symptom severity—⇓ QoL in all domains.	⇑ mutuality score (better caregiver–patient relationships)—⇑ QoL across all domains. Effective family coping strategies—⇑ QoL across all domains.
(Winahyu et al., 2015)	Indonesia Cross-sectional *n* = 137, 47.36 (12.22) 74.45% Outpatients	S-CGQoL	Employed caregivers—⇑ QoL (Beta = 0.15, *p* = 0.024). Gender, period of being a caregiver, and health status—no significant correlation with QoL.	n.s.	⇑ caregiver burden of care—⇓ QoL (Beta = −0.39, *p* < 0.001). ⇑ perceived social support—⇑⇑ QoL (Beta = 0.40, *p* < 0.001).
(Peng et al., 2024)	China Longitudinal *n* = 161, 66.32 (10.52) 49.07% Outpatients	WHOQoL-Bref	⇑ age—⇓ QoL (in the physical and psychological domains). ⇑ education levels—⇑ QoL (in the psychological domain). ⇑ income—⇑ QoL.	⇑ psychiatric symptom severity—⇓ QoL in all domains.	⇑ expressed emotion—⇓⇓ QoL. ⇑ internalized stigma—⇓⇓ QoL
(Caqueo-Urízar et al., 2016a)	Bolivia, Chile, Peru Cross-sectional *n* = 253, 54.7 (14.4), 67% Outpatients	S-CGQoL	Being female, older, unemployed, Aymara, and having a low family income—⇓ QoL levels.	n.s.	Greater perception of patient’s neurocognitive and social cognitive deficits—⇓ QoL in family relationships.
(Ghosh et al., 2020)	India Cross-sectional *n* = 35, 42.5 (12.1), 37.1% Patients’ setting n.s.	WHOQoL-Bref	n.s.	n.s.	⇑ caregiver burden of care—⇓ QoL in the psychological domain (*p* = 0.040) and the environmental domain (*p* = 0.030).

WHOQoL-Bref, World Health Organization Quality of Life—Abbreviated version; S-CGQoL, Schizophrenia Caregiver Quality of Life Questionnaire; SF-36, Short Form-36 Health Survey; SF-12 v1, Short Form-12 Health Survey, version 1; n, number; SD, standard deviation; QoL, quality of life; n.s., not specified; ⇑, increased; ⇓, decreased; ⇑⇑, marked increase; ⇓⇓, marked decrease; ♀, female gender; ♂, male gender.

**Table 2:** Methodological quality assessment of the included studies.

Article	Study Design	Question Number								Overall Score	Methodological Quality
		1	2	3	4	5	6	7	8		
(Ribé et al., 2018)	cross-sectional	Y	Y	Y	Y	Y	Y	Y	Y	8 100%	excellent
(Stanley et al., 2017)	cross-sectional	Y	Y	Y	Y	N	N	Y	Y	6 75%	moderate
(Kate et al., 2014)	cross-sectional	Y	Y	U	Y	N	N	Y	Y	5 62.5%	moderate
(Margetić et al., 2013)	cross-sectional	Y	Y	U	Y	N	N	Y	Y	5 62.5%	moderate
(Mizuno et al., 2012)	cross-sectional	Y	Y	Y	Y	Y	Y	Y	Y	8 100%	excellent
(Quah, 2014)	cross-sectional	Y	Y	Y	Y	N	N	Y	Y	6 75%	moderate
(Kate et al., 2013b)	cross-sectional	Y	Y	Y	Y	N	N	Y	Y	6 75%	moderate
(Stanley & Balakrishnan, 2023)	cross-sectional	Y	Y	Y	Y	Y	Y	Y	Y	8 100%	excellent
(Khatimah et al., 2022)	cross-sectional	Y	Y	Y	Y	Y	Y	Y	Y	8 100%	excellent
(Tristiana et al., 2019)	cross-sectional	Y	Y	Y	Y	N	N	Y	Y	6 75%	moderate
(Francisquini et al., 2020)	cross-sectional	Y	Y	Y	Y	N	N	Y	Y	6 75%	moderate
(Caqueo-Urízar et al., 2016b)	cross-sectional	Y	Y	Y	Y	N	N	Y	Y	6 75%	moderate
(Caqueo-Urízar et al., 2016a)	cross-sectional	Y	Y	Y	Y	Y	Y	Y	Y	8 100%	excellent
(Khan & Ghourmulla Alghamdi, 2020)	cross-sectional	Y	Y	Y	Y	N	N	Y	Y	6 75%	moderate
(Geriani, 2015)	cross-sectional	Y	Y	Y	Y	N	N	Y	Y	6 75%	moderate
(Marutani et al., 2020)	cross-sectional	N	Y	Y	Y	Y	Y	Y	Y	7 87.5%	high
(Ukpong & Ibigbami, 2021)	cross-sectional	Y	Y	Y	Y	Y	Y	Y	Y	8 100%	excellent
(Wang et al., 2023)	cross-sectional	Y	Y	U	Y	N	N	Y	Y	5 62.5%	moderate
(Opoku-Boateng et al., 2017)	cross-sectional	N	Y	Y	Y	Y	Y	Y	Y	7 87.5%	high
(Yerriah et al., 2022)	cross-sectional	Y	Y	Y	Y	Y	Y	Y	Y	8 100%	excellent
(Boyer et al., 2012)	cross-sectional	Y	Y	Y	Y	Y	Y	Y	Y	8 100%	excellent
(Kate et al., 2013a)	cross-sectional	Y	Y	U	Y	N	N	Y	Y	5 62.5%	moderate
(Li et al., 2007)	cross-sectional	Y	Y	Y	Y	Y	Y	Y	Y	8 100%	excellent
(ZamZam et al., 2011)	cross-sectional	Y	Y	Y	Y	Y	Y	Y	Y	8 100%	excellent
(Vadher et al., 2020)	cross-sectional	Y	Y	Y	Y	N	N	Y	Y	6 75%	moderate
(Usman et al., 2021)	cross-sectional	Y	Y	Y	Y	N	N	Y	Y	6 75%	moderate
(Hsiao et al., 2020)	cross-sectional	Y	Y	Y	Y	Y	Y	Y	Y	8 100%	excellent
(Winahyu et al., 2015)	cross-sectional	Y	Y	Y	Y	Y	Y	Y	Y	8 100%	excellent
(Peng et al., 2024)	cross-sectional	Y	Y	Y	Y	N	N	Y	Y	6 75%	moderate
(Caqueo-Urízar et al., 2016b)	cross-sectional	Y	Y	Y	Y	N	N	Y	Y	6 75%	moderate
(Ghosh et al., 2020)	cross-sectional	Y	Y	Y	Y	N	N	Y	Y	6 75%	moderate

Y, yes; N, no; U, unclear. Q1 = Were the criteria for inclusion in the sample clearly defined? Q2 = Were the study subjects and the setting described in detail? Q3 = Was the exposure measured in a valid and reliable way? Q4 = Were objective standard criteria used for the measurement of the condition? Q5 = Were confounding factors identified? Q6 = Were strategies to deal with confounding factors stated? Q7 = Were the outcomes measured in a valid and reliable way? Q8 = Was appropriate statistical analysis used?

## Data Availability

The additional data from this review are not publicly available but can be obtained upon request from the corresponding author.

## Supplementary Materials

The following supporting information can be downloaded at https://www.mdpi.com/article/10.3390/bs15050684/s1, Full search strategy; Table S1: PRISMA 2020 Checklist; Table S2: Reasons for exclusion of articles after full-text assessment.

## Abbreviations

The following abbreviations are used in this manuscript:

PwS	patient(s) with schizophrenia
QoL	quality of life
PRISMA	Preferred Reporting Items for Systematic reviews and Meta-Analyses
PICO	Patient/Population, Intervention/Exposure, Comparison and Outcome
JBI	Joanna Briggs Institute
WHOQoL-Bref	World Health Organization Quality of Life scale—abbreviated version
Schizophrenia Caregiver Quality of Life Questionnaire	S-CGQoL
SF-36	Short Form-36 Health Survey
SF-12 v1	Short Form-12 Health Survey, version 1
SD	standard deviation
PANSS	Positive and Negative Syndrome Scale
RCT	Randomized Controlled Trial
GRADE	Grading of Recommendations Assessment, Development and Evaluation
