# Supplementary material for: Quality of Life in Caregivers of Patients with Schizophrenia: A Systematic Review of the Impact of Sociodemographic, Clinical, and Psychological Factors
Source: Behav Sci (Basel). 2025 May 17;15(5):684. doi: 10.3390/bs15050684 (PMC12109056; doi:10.3390/bs15050684)
Supplement: Supplementary file 1 [file behavsci-15-00684-s001.zip › behavsci-3589880-supplementary.pdf]

Gagiu C. et al. Quality of life in caregivers of patients with schizophrenia: a systematic review of the impact of socio-demographic, clinical and psychological factors (2025)

Supplementary Materials

Full search strategies

Scopus:
(TITLE-ABS-KEY (caregiver OR carer OR family OR relative) AND TITLE-ABS-KEY (schizo* OR psychosis) AND TITLE-ABS-KEY ({quality of life} OR {life satisfaction}))

PubMed:
Search: ("quality of life" OR "life satisfaction") AND (schizo* OR psychosis) AND (caregiver OR carer OR family OR relative)
("quality of life"[All Fields] OR "life satisfaction"[All Fields]) AND ("schizo*"[All Fields] OR ("psychotic disorders"[MeSH Terms] OR ("psychotic"[All Fields] AND "disorders"[All Fields]) OR "psychotic disorders"[All Fields] OR "psychosis"[All Fields])) AND ("caregiver s"[All Fields] OR "caregivers"[MeSH Terms] OR "caregivers"[All Fields] OR "caregiver"[All Fields] OR "caregiving"[All Fields] OR ("caregivers"[MeSH Terms] OR "caregivers"[All Fields] OR "carer"[All Fields] OR "carers"[All Fields] OR "carer s"[All Fields]) OR ("familialities"[All Fields] OR "familiality"[All Fields] OR "familially"[All Fields] OR "familials"[All Fields] OR "familie"[All Fields] OR "family"[MeSH Terms] OR "family"[All Fields] OR "familial"[All Fields] OR "families"[All Fields] OR "family s"[All Fields] OR "familys"[All Fields]) OR ("family"[MeSH Terms] OR "family"[All Fields] OR "relative"[All Fields] OR "relatives"[All Fields] OR "relative s"[All Fields] OR "relatively"[All Fields]))

Web of Science:
((ALL=(caregiver OR carer OR family OR relative)) AND ALL=(schizo* OR psychosis)) AND ALL=("quality of life" OR "life satisfaction")

Table S1
PRISMA 2020 Checklist

Section and Topic	Item #	Checklist item	Location where item is reported
TITLE			
Title	1	Identify the report as a systematic review.	1
ABSTRACT			
Abstract	2	See the PRISMA 2020 for Abstracts checklist.	1
INTRODUCTION			
Rationale	3	Describe the rationale for the review in the context of existing knowledge.	1-3
Objectives	4	Provide an explicit statement of the objective(s) or question(s) the review addresses.	3
METHODS			
Eligibility criteria	5	Specify the inclusion and exclusion criteria for the review and how studies were grouped for the syntheses.	4
Information sources	6	Specify all databases, registers, websites, organisations, reference lists and other sources searched or consulted to identify studies. Specify the date when each source was last searched or consulted.	3
Search strategy	7	Present the full search strategies for all databases, registers and websites, including any filters and limits used.	Supplementary material
Selection process	8	Specify the methods used to decide whether a study met the inclusion criteria of the review, including how many reviewers screened each record and each report retrieved, whether they worked independently, and if applicable, details of automation tools used in the process.	3-4
Data collection process	9	Specify the methods used to collect data from reports, including how many reviewers collected data from each report, whether they worked independently, any processes for obtaining or confirming data from study investigators, and if applicable, details of automation tools used in the process.	4
Data items	10a	List and define all outcomes for which data were sought. Specify whether all results that were compatible with each outcome domain in each study were sought (e.g. for all measures, time points, analyses), and if not, the methods used to decide which results to collect.	4
	10b	List and define all other variables for which data were sought (e.g. participant and intervention characteristics, funding sources). Describe any assumptions made about any missing or unclear information.	4

Section and Topic	Item #	Checklist item	Location where item is reported
Study risk of bias assessment	11	Specify the methods used to assess risk of bias in the included studies, including details of the tool(s) used, how many reviewers assessed each study and whether they worked independently, and if applicable, details of automation tools used in the process.	4
Effect measures	12	Specify for each outcome the effect measure(s) (e.g. risk ratio, mean difference) used in the synthesis or presentation of results.	-
Synthesis methods	13a	Describe the processes used to decide which studies were eligible for each synthesis (e.g. tabulating the study intervention characteristics and comparing against the planned groups for each synthesis (item #5)).	5
	13b	Describe any methods required to prepare the data for presentation or synthesis, such as handling of missing summary statistics, or data conversions.	-
	13c	Describe any methods used to tabulate or visually display results of individual studies and syntheses.	4
	13d	Describe any methods used to synthesize results and provide a rationale for the choice(s). If meta-analysis was performed, describe the model(s), method(s) to identify the presence and extent of statistical heterogeneity, and software package(s) used.	4
	13e	Describe any methods used to explore possible causes of heterogeneity among study results (e.g. subgroup analysis, meta-regression).	-
	13f	Describe any sensitivity analyses conducted to assess robustness of the synthesized results.	-
Reporting bias assessment	14	Describe any methods used to assess risk of bias due to missing results in a synthesis (arising from reporting biases).	4
Certainty assessment	15	Describe any methods used to assess certainty (or confidence) in the body of evidence for an outcome.	-
RESULTS			
Study selection	16a	Describe the results of the search and selection process, from the number of records identified in the search to the number of studies included in the review, ideally using a flow diagram.	5
	16b	Cite studies that might appear to meet the inclusion criteria, but which were excluded, and explain why they were excluded.	Supplementary material: Table S2
Study characteristics	17	Cite each included study and present its characteristics.	6-12

Section and Topic	Item #	Checklist item	Location where item is reported
Risk of bias in studies	18	Present assessments of risk of bias for each included study.	12-13
Results of individual studies	19	For all outcomes, present, for each study: (a) summary statistics for each group (where appropriate) and (b) an effect estimate and its precision (e.g. confidence/credible interval), ideally using structured tables or plots.	6-12
Results of syntheses	20a	For each synthesis, briefly summarise the characteristics and risk of bias among contributing studies.	12
	20b	Present results of all statistical syntheses conducted. If meta-analysis was done, present for each the summary estimate and its precision (e.g. confidence/credible interval) and measures of statistical heterogeneity. If comparing groups, describe the direction of the effect.	5-6, 13-17
	20c	Present results of all investigations of possible causes of heterogeneity among study results.	-
	20d	Present results of all sensitivity analyses conducted to assess the robustness of the synthesized results.	4
Reporting biases	21	Present assessments of risk of bias due to missing results (arising from reporting biases) for each synthesis assessed.	12-13
Certainty of evidence	22	Present assessments of certainty (or confidence) in the body of evidence for each outcome assessed.	-
DISCUSSION			
Discussion	23a	Provide a general interpretation of the results in the context of other evidence.	17-20
	23b	Discuss any limitations of the evidence included in the review.	20-21
	23c	Discuss any limitations of the review processes used.	20-21
	23d	Discuss implications of the results for practice, policy, and future research.	20
OTHER INFORMATION			
Registration and protocol	24a	Provide registration information for the review, including register name and registration number, or state that the review was not registered.	3
	24b	Indicate where the review protocol can be accessed, or state that a protocol was not prepared.	3
	24c	Describe and explain any amendments to information provided at registration or in the protocol.	-
Support	25	Describe sources of financial or non-financial support for the review, and the role of the funders or sponsors in the review.	22
Competing	26	Declare any competing interests of review authors.	22

Section and Topic	Item #	Checklist item	Location where item is reported
interests			
Availability of data, code and other materials	27	Report which of the following are publicly available and where they can be found: template data collection forms; data extracted from included studies; data used for all analyses; analytic code; any other materials used in the review.	22

Table S2

Reasons for exclusion of articles after full-text assessment

No.	Article	Reason for exclusion
1	Jones CJ, Hazell CM, Hayward M, et al. Caring for Carers (C4C): Results from a feasibility randomised controlled trial of positive written disclosure for older adult carers of people with psychosis. *PLoS One*. 2022;17(11):e0277196. doi:10.1371/journal.pone.0277196	No relationship between QoL and other variables
2	Gonçalves-Pereira M, González-Fraile E, Santos-Zorrozúa B, et al. Assessment of the consequences of caregiving in psychosis: a psychometric comparison of the Zarit Burden Interview (ZBI) and the Involvement Evaluation Questionnaire (IEQ). *Health Qual Life Outcomes*. 2017;15(1):63. doi:10.1186/s12955-017-0626-8	No relationship between QoL and other variables
3	Rhee TG, Rosenheck RA. Does improvement in symptoms and quality of life in chronic schizophrenia reduce family caregiver burden?. *Psychiatry Res*. 2019;271:402-404. doi:10.1016/j.psychres.2018.12.005	Articles that did not assessed QoL
4	Koenig M, Castillo MC, Urdapilleta I, Le Borgne P, Bouleau JH. Regards croisés sur les signes précoces de rechute des sujets schizophrènes [Relapse in schizophrenia: an exploratory study of the joint conceptions of patients, parents and caregivers]. *Encephale*. 2011;37(3):207-216. doi:10.1016/j.encep.2010.06.002	Other languages (French)
5	Çırakman D, Karslıoğlu EH, Bal NB, Çayköylü A. The effect of early or late initiation of long-acting antipsychotics on the caregiver burden in schizophrenia. *Int Clin Psychopharmacol*. 2024;39(5):313-322. doi:10.1097/YIC.0000000000000524	No relationship between QoL and other variables
6	Hayes L, Hawthorne G, Farhall J, O'Hanlon B, Harvey C. Quality of Life and Social Isolation Among Caregivers of Adults with Schizophrenia: Policy and Outcomes. *Community Ment Health J*. 2015;51(5):591-597. doi:10.1007/s10597-015-9848-6	No relationship between QoL and other variables
7	Sara Sapharina GJ, Neelakshi G. Effectiveness of psychoeducative intervention on quality of life among caregivers of patients with schizophrenia: A randomized control trial. *Indian J Public Health*. 2022;66(4):439-442. doi:10.4103/ijph.ijph_2094_21	No relationship between QoL and other variables

8	Foster K. 'You'd think this roller coaster was never going to stop': experiences of adult children of parents with serious mental illness. *J Clin Nurs*. 2010;19(21-22):3143-3151. doi:10.1111/j.1365-2702.2010.03293.x	Qualitative study
9	Alkan O, Kushnir J, Bar M, Kushnir T. Quality of life of adult daughters of women with schizophrenia: Associations with psychological resource losses and gains. *Compr Psychiatry*. 2016;68:11-17. doi:10.1016/j.comppsych.2016.03.002	Not caregivers
10	Lueboonthavatchai P, Lueboonthavatchai O. Quality of life and correlated health status and social support of schizophrenic patients' caregivers. *J Med Assoc Thai*. 2006;89 Suppl 3:S13-S19.	No relationship between QoL and other variables
11	Csoboth C, Witt EA, Villa KF, O'Gorman C. The humanistic and economic burden of providing care for a patient with schizophrenia. *Int J Soc Psychiatry*. 2015;61(8):754-761. doi:10.1177/0020764015577844	Articles that did not assessed QoL
12	Perlick DA, Rosenheck RA, Kaczynski R, Swartz MS, Cañive JM, Lieberman JA. Components and correlates of family burden in schizophrenia. *Psychiatr Serv*. 2006;57(8):1117-1125. doi:10.1176/ps.2006.57.8.1117	Articles that did not assessed QoL
13	Hasan AA, Callaghan P, Lymn JS. Evaluation of the impact of a psycho-educational intervention for people diagnosed with schizophrenia and their primary caregivers in Jordan: a randomized controlled trial. *BMC Psychiatry*. 2015;15:72. Published 2015 Apr 8. doi:10.1186/s12888-015-0444-7	No relationship between QoL and other variables
14	Hasan AA, Callaghan P, Lymn JS. Evaluation of the impact of a psycho-educational intervention on knowledge levels and psychological outcomes for people diagnosed with Schizophrenia and their caregivers in Jordan: a randomized controlled trial. *BMC Psychiatry*. 2014;14:17. Published 2014 Jan 22. doi:10.1186/1471-244X-14-17	No relationship between QoL and other variables
15	Caqueo-Urízar A, Gutiérrez-Maldonado J. Burden of care in families of patients with schizophrenia. *Qual Life Res*. 2006;15(4):719-724. doi:10.1007/s11136-005-4629-2	Articles that did not assessed QoL
16	Rofail D, Regnault A, le Scouiller S, Lambert J, Zarit SH. Assessing the impact on caregivers of patients with schizophrenia: psychometric validation of the Schizophrenia	Articles that did not assessed QoL

	Caregiver Questionnaire (SCQ). *BMC Psychiatry*. 2016;16:245. Published 2016 Jul 18. doi:10.1186/s12888-016-0951-1	
17	Grandón P, Jenaro C, Lemos S. Primary caregivers of schizophrenia outpatients: burden and predictor variables. *Psychiatry Res*. 2008;158(3):335-343. doi:10.1016/j.psychres.2006.12.013	Articles that did not assessed QoL
18	Kasperek-Zimowska B, Chadzyńska M. Poczucie koherencji i style radzenia sobie ze stresem wśród rodziców dorosłych dzieci z rozpoznaniem schizofrenii [Sense of coherence and coping styles among parents of adult children with schizophrenia]. Psychiatr Pol. 2011;45(5):643-652.	Other languages (Polish)
19	Gupta S, Isherwood G, Jones K, Van Impe K. Assessing health status in informal schizophrenia caregivers compared with health status in non-caregivers and caregivers of other conditions. *BMC Psychiatry*. 2015;15:162. doi:10.1186/s12888-015-0547-1	No relationship between QoL and other variables
20	Parabiaghi A, Lasalvia A, Bonetto C, et al. Predictors of changes in caregiving burden in people with schizophrenia: a 3-year follow-up study in a community mental health service. *Acta Psychiatr Scand Suppl*. 2007;(437):66-76. doi:10.1111/j.1600-0447.2007.01094.x	Articles that did not assessed QoL
21	Richieri R, Boyer L, Reine G, et al. Validation française d'un questionnaire de qualité de vie des aidants naturels de patients schizophrènes [A preliminary validation of a new French instrument to assess quality of life for caregivers of patients suffering from schizophrenia]. *Encephale*. 2011;37(6):425-432. doi:10.1016/j.encep.2011.01.005	Other languages (French)
22	Alpi A, Cocchi A, Meneghelli A, Pafumi N, Patelli G. Lavorare con le famiglie negli esordi psicotici: un intervento strutturato per i caregiver [Working with families in the early stages of psychosis: a structured intervention for caregivers]. *G Ital Med Lav Ergon*. 2008;30(3 Suppl B):B62-B70.	Other languages (Italian)
23	Foldemo A, Gullberg M, Ek AC, Bogren L. Quality of life and burden in parents of outpatients with schizophrenia. *Soc Psychiatry Psychiatr Epidemiol*. 2005;40(2):133-138. doi:10.1007/s00127-005-0853-x	Not caregivers

24	Allerby K, Sameby B, Brain C, et al. Stigma and Burden Among Relatives of Persons With Schizophrenia: Results From the Swedish COAST Study. *Psychiatr Serv*. 2015;66(10):1020-1026. doi:10.1176/appi.ps.201400405	Articles that did not assessed QoL
25	Kochhar SS, Mishra AK, Chadda RK, Sood M, Bhargava R. Changes in Psychosocial Variables Among Caregivers of Patients With Schizophrenia: A Short-Term Follow-Up Study. *Cureus*. 2024;16(3):e55887. doi:10.7759/cureus.55887	Articles that did not assessed QoL
26	Wei Y, Peng Y, Li Y, Song L, Ju K, Xi J. Caregivers' burden and schizophrenia patients' quality of life: Sequential mediating effects of expressed emotion and perceived expressed emotion. *Front Psychiatry*. 2022;13:961691. doi:10.3389/fpsyt.2022.961691	Articles that did not assessed QoL
27	Tessier A, Roger K, Gregoire A, Desnavailles P, Misdrahi D. Family psychoeducation to improve outcome in caregivers and patients with schizophrenia: a randomized clinical trial. *Front Psychiatry*. 2023;14:1171661. Published 2023 Jun 23. doi:10.3389/fpsyt.2023.1171661	No relationship between QoL and other variables
28	Chaturvedi SK, Hamza A, Sharma MP. Changes in distressing behavior perceived by family of persons with schizophrenia at home - 25 years later. *Indian J Psychol Med*. 2014;36(3):282-287. doi:10.4103/0253-7176.135381	Articles that did not assessed QoL
29	Verma PK, Walia TS, Chaudhury S, Srivastava S. Family psychoeducation with caregivers of schizophrenia patients: Impact on perceived quality of life. *Ind Psychiatry J*. 2019;28(1):19-23. doi:10.4103/ipj.ipj_2_19	No relationship between QoL and other variables
30	Omranifard V, Yari A, Kheirabadi GR, Rafizadeh M, Maracy MR, Sadri S. Effect of needs-assessment-based psychoeducation for families of patients with schizophrenia on quality of life of patients and their families: A controlled study. *J Educ Health Promot*. 2014;3:125. Published 2014 Nov 29. doi:10.4103/2277-9531.145937	No relationship between QoL and other variables
31	Albikawi Z, Abuadas M. Quality of life and self-stigma of schizophrenia patient's caregiver tool: Development and validation using classical test theory and Rasch analysis. *S Afr J Psychiatr*. 2021;27:1656. doi:10.4102/sajpsychiatry.v27i0.1656	The scale is not validated

32	Kumar R, Nischal A, Dalal PK, et al. Impact of brief psychosocial intervention on key relatives of patients with schizophrenia: A randomized controlled trial. *Indian J Psychiatry*. 2020;62(2):137-144. doi:10.4103/psychiatry.IndianJPsychiatry_138_19	No relationship between QoL and other variables
33	Sustrami, D.; Suhardiningsih, A. S. .; Purbasari, D. N. .; Budiarti, A. A Descriptive Study of Quality of Life of Caregiver in the Family of Schizophrenia Patients. *The Malaysian Journal of Nursing* **2022**, *14*, 124-127. doi:10.31674/mjn.2022.v14i02.020	No relationship between QoL and other variables
34	Zelenkova TV, Konoreva AE. Perceptual-emotional technique of facilitating the psychotherapeutic process in working with relatives of schizophrenic patients. Psychiatry Psychotherapy and Clinical Psychology. 2022 Jun;13(2):16–25. doi:10.34883/PI.2022.13.2.002.	Other languages (Russian)
35	Vinent FR, Torres DC, Ortiz RML, Palay MDR, Columbié AH. Psycho-educational intervention strategy for relatives of patients with schizophrenic disorders. Rev Hosp Psiquiátrico Habana. 2020;17(3).	Other languages (Spanish)
36	Singh, Shipra; Sinha, Deoraj1,; Raut, Nitin B.2. Caregiving Experience and Marital Adjustment in Spouses of Patients with Schizophrenia. Indian Journal of Social Psychiatry 35(2):p 125-130, Apr–Jun 2019. | DOI: 10.4103/ijsp.ijsp_49_18	Articles that did not assessed QoL
37	Ferliana H, Damayanti NA, Aisyah DN, Huda N, Ernawati D. Determinants of family independence in caring for hebephrenic schizophrenia patients. J Public Health Res. 2020 Jul 2;9(2):1828. doi: 10.4081/jphr.2020.1828.	Articles that did not assessed QoL
38	LI Shiming,YANG Queping,ZHANG Zijuan, et al. Effect of Comprehensive Care and Support Interventions on the Psychological Status and Quality of Life of Family Caregivers of Community-dwelling Schizophrenic Patients[J]. Chinese General Practice, 2019, 22(4): 490-494. DOI: 10.12114/j.issn.1007-9572.2019.04.024.	Other languages (Chinese)
39	Boyer, L., Baumstarck, K., Auquier, P. (2016). Assessment of the Burden of Care and Quality of Life of Caregivers in Schizophrenia. In: Awad, A., Voruganti, L. (eds) Beyond Assessment of Quality of Life in Schizophrenia. Adis, Cham. https://doi.org/10.1007/978-3-319-30061-0_6	Abstract presented at conferences meeting, book chapters, literature reviews

40	Fan YC, Chen MB, Lin KC, Bai YM, Wei SJ. The resilience and health status of primary caregivers of schizophrenia patients. Hu Li Za Zhi. 2014;61(6):29-38. doi:10.6224/JN.61.6.29	Other languages (Chinese)
41	Bankovská Motlová L, Dragomirecká E, Čermák J, Čermáková R, Španiel F. Family psychoeducation for schizophrenia: Demographic characteristics and quality of life of relatives. Psychiatrie. 2009;13(4):136–	Abstract presented at conferences meeting, book chapters, literature reviews
42	Richieri R, Boyer L, Reine G, Loundou AD, Simeoni MC, Auquier P, et al. [A preliminary validation of a new French instrument to assess quality of life for caregivers of patients suffering from schizophrenia]. Encephale. 2011;37(6):425–32. doi:10.1016/j.encep.2011.01.005.	Other languages (French)
43	Luo N, Seng BK, Li SC. Identification of factors influencing caregiving experience in caregivers of patients with schizophrenia. *Ann Acad Med Singap*. 2003;32(5 Suppl):S43-S45.	Abstract presented at conferences meeting, book chapters, literature reviews
44	Jungbauer J, Angermeyer MC. Living with a schizophrenic patient: a comparative study of burden as it affects parents and spouses. *Psychiatry*. 2002;65(2):110-123. doi:10.1521/psyc.65.2.110.19930	Articles that did not assessed QoL
45	Rosenheck R, Cramer J, Jurgis G, et al. Clinical and psychopharmacologic factors influencing family burden in refractory schizophrenia. The Department of Veterans Affairs Cooperative Study Group on Clozapine in Refractory Schizophrenia. *J Clin Psychiatry*. 2000;61(9):671-676. doi:10.4088/jcp.v61n0913	Articles that did not assessed QoL
46	Bogren, L. Y. (1997). Expressed emotion, family burden, and quality of life in parents with schizophrenic children. *Nordic Journal of Psychiatry*, *51*(4), 229–233. https://doi.org/10.3109/08039489709090713	Not caregivers
47	Karaağaç H, Çalık Var E. Investigation of the effect between care burden and quality of life in caregivers of schizophrenia patients. Turk J Clin Psychopharmacol. 2019;22(1):16–26. doi:10.5505/kpd.2018.60783.	Other languages (Turkish)

48	Bequis-Lacera M, Munoz-Hernandez Y, Duque-Rojas O, Guzman-Quintero A, Numpaque-Molina A, Rojas-Gonzalez A, et al. Overload and quality of life of the caregiver of a patient with schizophrenia. Duazary. 2019;16(2):280–92. doi:10.21676/2389783X.2960.	Other languages (Spanish)
49	Suharsono, S., Faidah, N., & Hanafi, M. (2023). The effectiveness of nursing psychoeducation toward family burden and quality life on caregiver of people with schizophrenia in the community . Healthcare in Low-Resource Settings, 11(s1). https://doi.org/10.4081/hls.2023.11215	No relationship between QoL and other variables
50	McWilliams S, Hill S, Mannion N, Fetherston A, Kinsella A, O'Callaghan E. Schizophrenia: a five-year follow-up of patient outcome following psycho-education for caregivers. Eur Psychiatry. 2012;27(1):56-61. doi:10.1016/j.eurpsy.2010.08.012	Articles that did not assessed QoL
51	Norheim I, Pedersen R, Selle ML, Røssberg JI, Hestmark L, Heiervang KS, Ruud T, Åsholt VM, Hansson KM, Møller P, Fosse R and Romøren M (2024) Implementation of guidelines on Family Involvement for persons with Psychotic disorders: a pragmatic cluster randomized trial. Effect on relatives' outcomes and family interventions received. Front. Psychiatry 15:1381007. doi: 10.3389/fpsyt.2024.1381007	Other mental disorders
52	Ishaque, F. ., Emad, F. ., Masooma, S. ., Hasan, S. M. ., Zaidi, S. M. Z. ., & Azmat, S. F. . (2024). Sociodemographic Association of Caregivers Burden with Schizophrenia Disorder. Bangladesh Journal of Medical Science, 23(1), 89–94. https://doi.org/10.3329/bjms.v23i1.70691	Articles that did not assessed QoL
53	Nergiz Z, Günaydın N. Determining the Family Sense of Coherence and Caregiving Burden of Caregivers of Patients with Schizophrenia: A-Cross Sectional Study. JBACHS. May 2022;6(2):637-649. doi:10.30621/jbachs.993946	No relationship between QoL and other variables
54	Rahmani F, Roshangar F, Gholizadeh L, Asghari E. Caregiver burden and the associated factors in the family caregivers of patients with schizophrenia. Nurs Open. 2022 Jul;9(4):1995-2002. doi: 10.1002/nop2.1205.	Articles that did not assessed QoL
55	Roșca EA, Alexinschi O, Brîncuș C, Matei VP, Giurgiuca A. Quality of life in Romanian patients with schizophrenia based on gender, type of schizophrenia, therapeutic	Articles that did not assessed QoL

	approach, and family history. J Mind Med Sci. 2018; 5(2): 202-209. DOI: 10.22543/7674.52.P202209	
56	Thakurdesai A, Fernandes A, Merchant H, Nayak A, Parkar S. A study of burden and quality of life in caregivers of patients with schizophrenia. Indian J Psychiatry. 2015;57(Suppl 5):S91.	Abstract presented at conferences meeting, book chapters, literature reviews
57	Guan Z, Huang C, Wiley JA, Sun M, Bai X, Tang S. Internalized stigma and its correlates among family caregivers of patients diagnosed with schizophrenia in Changsha, Hunan, China. *J Psychiatr Ment Health Nurs*. 2020;27(3):224-236. doi:10.1111/jpm.12571	Articles that did not assessed QoL
58	Yu Y, Liu ZW, Li TX, Li YL, Xiao SY, Tebes JK. Test of the stress process model of family caregivers of people living with schizophrenia in China. *Soc Sci Med*. 2020;259:113113. doi:10.1016/j.socscimed.2020.113113	Articles that did not assessed QoL
59	Yu W, Chen J, Hu J, Hu J. Relationship between Mental Health and Burden among Primary Caregivers of Outpatients with Schizophrenia. *Fam Process*. 2019;58(2):370-383. doi:10.1111/famp.12340	Articles that did not assessed QoL
60	Lök N, Bademli K, Lök S. The effect of a physical activity intervention on burden and healthy lifestyle behavior in family caregivers of patients with schizophrenia: A randomized controlled trial. *Arch Psychiatr Nurs*. 2023;42:33-39. doi:10.1016/j.apnu.2022.12.006	Articles that did not assessed QoL
61	Lauber C, Eichenberger A, Luginbühl P, Keller C, Rössler W. Determinants of burden in caregivers of patients with exacerbating schizophrenia. *Eur Psychiatry*. 2003;18(6):285-289. doi:10.1016/j.eurpsy.2003.06.004	Articles that did not assessed QoL
62	Çiğdem Dereboy, Gözde Sayın Karakaş, Gamze Karadayı Kaynak, İlknur Kocadurdu, Ayşe Döndü, Fatma Demirkıran. The effects of ?I can ride the storm? program in functionality, life satisfaction, and perceived family burden in patients with schizophrenia. J Psychiatric Nurs 2023;14(1):33-41. DOI: 10.14744/phd.2022.29494	Articles that did not assessed QoL

63	Arslantas H, Adana F. Factors affecting caregivers' burden and emotional expression of patients with schizophrenia. Anadolu Psikiyatri Derg. 2012;13(1):8–15.	Other languages (Turkish)
64	Krautgartner M, Unger A, Göessler R, et al. Minderjährige Angehörige von Schizophrenie-Kranken: Belastungen und Unterstützungsbedarf [Minor relatives of schizophrenia patients: burden and needs]. Neuropsychiatr. 2007;21(4):267-274.	Other languages (German)
65	Schmid R, Schielein T, Binder H, Hajak G, Spiessl H. The forgotten caregivers: Siblings of schizophrenic patients. *Int J Psychiatry Clin Pract*. 2009;13(4):326-337. doi:10.3109/13651500903141400	Articles that did not assessed QoL
66	Peng MM, Ma Z, Ran MS. Family caregiving and chronic illness management in schizophrenia: positive and negative aspects of caregiving. *BMC Psychol*. 2022;10(1):83. Published 2022 Mar 31. doi:10.1186/s40359-022-00794-9	Qualitative study
67	Caqueo-Urízar A, Gutiérrez-Maldonado J, Ferrer-García M, Peñaloza-Salazar C, Richards-Araya D, Cuadra-Peralta A. Attitudes and burden in relatives of patients with schizophrenia in a middle income country. *BMC Fam Pract*. 2011;12(1):101. doi:10.1186/1471-2296-12-101	Articles that did not assessed QoL
68	Debsikdar SS, Patil NP, Gaikwad SJ. Quality of life, enjoyment and satisfaction in caregivers of patients with schizophrenia. Indian J Psychiatry. 2018 Feb;60(5):107.	Abstract presented at conferences meeting, book chapters, literature reviews
69	Di Lorenzo R, Girone A, Panzera N, et al. Empathy and perceived burden in caregivers of patients with schizophrenia spectrum disorders. *BMC Health Serv Res*. 2021;21(1):250. doi:10.1186/s12913-021-06258-x	Articles that did not assessed QoL
70	Kidd SA, Kerman N, Ernest D, et al. A pilot study of a family cognitive adaptation training guide for individuals with schizophrenia. *Psychiatr Rehabil J*. 2018;41(2):109-117. doi:10.1037/prj0000204	Articles that did not assessed QoL
71	Zang D, Zhang XT, Li YL, Li TX, Xiao SY, Tebes JK, Yu Y. WeChat use among family caregivers of people living with schizophrenia and its relationship to caregiving experiences. Comput Hum Behav. 2021 Oct;123:106877.	Articles that did not assessed QoL

72	Durmaz H, Okanlı A. Investigation of the effect of self-efficacy levels of caregiver family members of the individuals with schizophrenia on burden of care. *Arch Psychiatr Nurs*. 2014;28(4):290-294. doi:10.1016/j.apnu.2014.04.004	Articles that did not assessed QoL
73	Ata EE, Doğan S. The Effect of a Brief Cognitive Behavioural Stress Management Programme on Mental Status, Coping with Stress Attitude and Caregiver Burden While Caring for Schizophrenic Patients. *Arch Psychiatr Nurs*. 2018;32(1):112-119. doi:10.1016/j.apnu.2017.10.004	Articles that did not assessed QoL
74	Fischer M, Kemmler G, Meise U. "Schön, dass sich auch einmal jemand für mich interessiert" - Eine Erhebung der Lebensqualität von Angehörigen langzeitig an Schizophrenie Erkrankter ["How Nice that Someone is Interested in me for a Change"]. *Psychiatr Prax*. 2004;31(2):60-67. doi:10.1055/s-2003-814797	Other languages (Turkish)
75	Richieri R, Boyer L, Reine G, et al. The Schizophrenia Caregiver Quality of Life questionnaire (S-CGQoL): development and validation of an instrument to measure quality of life of caregivers of individuals with schizophrenia. *Schizophr Res*. 2011;126(1-3):192-201. doi:10.1016/j.schres.2010.08.037	The scale is not validated
76	Sikirica V, Markowitz JS, Engelhart LM. Schizophrenia caregiver burden and correlates to health-related quality of life. Qual Life Res. 2005 Nov;14(9):2000.	Abstract presented at conferences meeting, book chapters, literature reviews
77	Bagheri S, Zarshenas L, Rakhshan M, et al. Impact of Watson's human caring-based health promotion program on caregivers of individuals with schizophrenia [published correction appears in BMC Health Serv Res. 2023 Jul 13;23(1):748. doi: 10.1186/s12913-023-09800-1]. *BMC Health Serv Res*. 2023;23(1):711. doi:10.1186/s12913-023-09725-9	Articles that did not assessed QoL
78	Kamizawa, Naotoshi & Miyamura, Toshihiro. (2023). Translation and validation of the Schizophrenia Caregiver Questionnaire – Japanese version (J-SCQ). Heliyon. 9. e13338. 10.1016/j.heliyon.2023.e13338.	The scale is not validated

79	Lök N, Bademli K. The Relationship Between the Perceived Social Support and Psychological Resilience in Caregivers of Patients with Schizophrenia. *Community Ment Health J*. 2021;57(2):387-391. doi:10.1007/s10597-020-00665-w	Articles that did not assessed QoL
80	Pitschel-Walz G, Rummel-Kluge C, Froboese T, Beitinger R, Stiegler M, Baeuml J, Kissling W. Enhancing empowerment for relatives of patients with schizophrenia. Results of a psychoeducational group program under naturalistic conditions. Psychother Psychosom Med Psychol. 2012 Jul;57(4):313-8. doi: 10.1007/s00278-012-0924-x.	Other languages (Turkish)
81	Young L, Digel Vandyk A, Daniel Jacob J, McPherson C, Murata L. Being Parent Caregivers for Adult Children with Schizophrenia. *Issues Ment Health Nurs*. 2019;40(4):297-303. doi:10.1080/01612840.2018.1524531	Qualitative study
82	Bulut M, Arslantaş H, Ferhan Dereboy İ. Effects of Psychoeducation Given to Caregivers of People With a Diagnosis of Schizophrenia. *Issues Ment Health Nurs*. 2016;37(11):800-810. doi:10.1080/01612840.2016.1222039	Articles that did not assessed QoL
83	Yu Y, Liu ZW, Tang BW, Zhao M, Liu XG, Xiao SY. Reported family burden of schizophrenia patients in rural China. *PLoS One*. 2017;12(6):e0179425. doi:10.1371/journal.pone.0179425	Articles that did not assessed QoL
84	Gater A, Rofail D, Marshall C, et al. Assessing the Impact of Caring for a Person with Schizophrenia: Development of the Schizophrenia Caregiver Questionnaire. *Patient*. 2015;8(6):507-520. doi:10.1007/s40271-015-0114-3	Abstract presented at conferences meeting, book chapters, literature reviews
85	Sharifi M, Younesi SJ, Foroughan M, Safi MH, Khanjani MS. The Challenges of Caring for an Adult Child with Schizophrenia in the Family: An Analysis of the Lived Experiences of Older Parents. *Inquiry*. 2023;60:469580221148867. doi:10.1177/00469580221148867	Qualitative study
86	Sahin F, Altun ÖŞ. The relationship between perceived family support and happiness level of patients with schizophrenia. J Psychiatr Nurs. 2020 Jan;11(3):181-7. doi: 10.14744/phd.2020.09821.	Articles that did not assessed QoL

87	Bademli K, Lök N, Kılıç AK. The Relationship Between the Burden of Caregiving, Submissive Behaviors and Depressive Symptoms in Primary Caregivers of Patients With Schizophrenia. *Arch Psychiatr Nurs*. 2018;32(2):229-234. doi:10.1016/j.apnu.2017.11.007	Articles that did not assessed QoL
88	Memon, Muhammad Raza & Ali, Manzoor & Chachar, Qasim & Shaikh, Bhaktawar & Ahmed, Zuhaib & Ansari, Moin. (2023). Psychological distress among family care givers of people with mental illness at Hyderabad, Pakistan. Rawal Medical Journal. 48. 204-208.	Other mental disorders
89	Alexander G, Bebee CE, Chen KM, et al. Burden of caregivers of adult patients with schizophrenia in a predominantly African ancestry population [published correction appears in Qual Life Res. 2019, 28(5):1387. doi: 10.1007/s11136-018-02096-3	Articles that did not assessed QoL
90	de la Serna E, Baeza I, Toro J, et al. Relationship between clinical and neuropsychological characteristics in child and adolescent first degree relatives of subjects with schizophrenia. *Schizophr Res*. 2010;116(2-3):159-167. doi:10.1016/j.schres.2009.09.001	Pediatric patients
91	Knock J, Kline E, Schiffman J, Maynard A, Reeves G. Burdens and difficulties experienced by caregivers of children and adolescents with schizophrenia-spectrum disorders: a qualitative study. *Early Interv Psychiatry*. 2011;5(4):349-354. doi:10.1111/j.1751-7893.2011.00305.x	Pediatric patients
92	Möller-Leimkühler, A. M., & Obermeier, M. (2008). Predicting caregiver burden in first admission psychiatric patients. 2-year follow-up results. European archives of psychiatry and clinical neuroscience, 258(7), 406–413. https://doi.org/10.1007/s00406-008-0818-7	Articles that did not assessed QoL
93	Stanley, S., & Balakrishnan, S. (2023). Informal caregivers of people with a diagnosis of schizophrenia: determinants and predictors of resilience. Journal of mental health (Abingdon, England), 32(1), 198–205. https://doi.org/10.1080/09638237.2021.1952945	Articles that did not assessed QoL
94	Pietrini, F., Tatini, L., Santarelli, G., Brugnolo, D., Squillace, M., Bozza, B., Ballerini, A., Ricca, V., & D'Anna, G. (2021). Self- and caregiver-perceived disability, subjective well-	Articles that did not assessed QoL

	being, quality of life and psychopathology improvement in long-acting antipsychotic treatments: a 2-year follow-up study. International journal of psychiatry in clinical practice, 25(3), 307–315. https://doi.org/10.1080/13651501.2021.1912358	
95	Gómez-de-Regil, L., Kwapil, T. R., & Barrantes-Vidal, N. (2014). Predictors of expressed emotion, burden and quality of life in relatives of Mexican patients with psychosis. Journal of psychiatric and mental health nursing, 21(2), 170–179. https://doi.org/10.1111/jpm.12071	Other mental disorders
96	Alptekin, K., Akdede, B. B., Akvardar, Y., Celikgün, S., Dilşen, N. S., Durak, G., Türk, A., & Fidaner, H. (2004). Quality of life assessment in Turkish patients with schizophrenia and their relatives. Psychological reports, 95(1), 197–206. https://doi.org/10.2466/pr0.95.1.197-206	Articles that did not assessed QoL
97	Möller-Leimkühler, A. M., & Wiesheu, A. (2012). Caregiver burden in chronic mental illness: the role of patient and caregiver characteristics. European archives of psychiatry and clinical neuroscience, 262(2), 157–166. https://doi.org/10.1007/s00406-011-0215-5	Other mental disorders
98	Zahid, M. A., & Ohaeri, J. U. (2010). Relationship of family caregiver burden with quality of care and psychopathology in a sample of Arab subjects with schizophrenia. BMC psychiatry, 10, 71. https://doi.org/10.1186/1471-244X-10-71	Articles that did not assessed QoL
99	Martens, L., & Addington, J. (2001). The psychological well-being of family members of individuals with schizophrenia. Social psychiatry and psychiatric epidemiology, 36(3), 128–133. https://doi.org/10.1007/s001270050301	Articles that did not assessed QoL
100	Rungreangkulkij, S., Chafetz, L., Chesla, C., & Gilliss, C. (2002). Psychological morbidity of Thai families of a person with schizophrenia. International journal of nursing studies, 39(1), 35–50. https://doi.org/10.1016/s0020-7489(01)00005-0	Articles that did not assessed QoL
101	Chien, W. T., Chan, S. W., & Morrissey, J. (2007). The perceived burden among Chinese family caregivers of people with schizophrenia. Journal of clinical nursing, 16(6), 1151–1161. https://doi.org/10.1111/j.1365-2702.2007.01501.x	Articles that did not assessed QoL
102	Kadri, N., Manoudi, F., Berrada, S., & Moussaoui, D. (2004). Stigma impact on Moroccan families of patients with schizophrenia. Canadian journal of psychiatry.	Articles that did not assessed QoL

	Revue canadienne de psychiatrie, 49(9), 625–629. https://doi.org/10.1177/070674370404900909	
103	Möller-Leimkühler A. M. (2006). Multivariate prediction of relatives' stress outcome one year after first hospitalization of schizophrenic and depressed patients. European archives of psychiatry and clinical neuroscience, 256(2), 122–130. https://doi.org/10.1007/s00406-005-0619-1	Other mental disorders
104	Jungbauer, J., Wittmund, B., Dietrich, S., & Angermeyer, M. C. (2004). The disregarded caregivers: subjective burden in spouses of schizophrenia patients. Schizophrenia bulletin, 30(3), 665–675. https://doi.org/10.1093/oxfordjournals.schbul.a007114	Qualitative study
105	Ehsan, N., Johar, N., Saleem, T., Khan, M. A., & Ghauri, S. (2018). Negative repercussions of caregiving burden: Poor psychological well-being and depression. Pakistan journal of medical sciences, 34(6), 1452–1456. https://doi.org/10.12669/pjms.346.15915	Articles that did not assessed QoL
106	Uysal, F., Yildizhan, E., & Tomruk, N. B. (2022). Relationship of Long Acting Injectable Antipsychotics with Caregiver Burden, Quality of Life, Symptom Severity and Treatment Discontinuation in Schizophrenia. Şizofrenide Uzun Etkili Enjekte Edilebilir Antipsikotiklerin Bakım Veren Yükü, Yaşam Kalitesi, Belirti Şiddeti ve Tedaviyi Bırakma ile İlişkisi. Turk psikiyatri dergisi = Turkish journal of psychiatry, 33(3), 167–179. https://doi.org/10.5080/u26066	Articles that did not assessed QoL
107	Awadalla, A. W., Ohaeri, J. U., Salih, A. A., & Tawfiq, A. M. (2005). Subjective quality of life of community living Sudanese psychiatric patients: comparison with family caregivers' impressions and control group. Quality of life research : an international journal of quality of life aspects of treatment, care and rehabilitation, 14(8), 1855–1867. https://doi.org/10.1007/s11136-005-4328-z	Articles that did not assessed QoL
108	Zhou, Z., Wang, Y., Feng, P., Li, T., Tebes, J. K., Luan, R., & Yu, Y. (2021). Associations of Caregiving Knowledge and Skills With Caregiver Burden, Psychological Well-Being, and Coping Styles Among Primary Family Caregivers of People Living With	Articles that did not assessed QoL

	Schizophrenia in China. Frontiers in psychiatry, 12, 631420. https://doi.org/10.3389/fpsyt.2021.631420	
109	Fe Bravo-Ortiz, M., Gutiérrez-Casares, J. R., Rodríguez-Morales, A., García, M. A., & Hidalgo-Borrajo, R. (2011). Influence of type of treatment on the well-being of Spanish patients with schizophrenia and their caregivers. International journal of psychiatry in clinical practice, 15(4), 286–295. https://doi.org/10.3109/13651501.2011.608469	Articles that did not assessed QoL
110	Brissos, S., Afonso, P., Cañas, F., Bobes, J., Bernardo Fernandez, I., & Guzman, C. (2013). Satisfaction with Life of Schizophrenia Outpatients and Their Caregivers: Differences between Patients with and without Self-Reported Sleep Complaints. Schizophrenia research and treatment, 2013, 502172. https://doi.org/10.1155/2013/502172	The scale is not validated
